# Selection strategies to introgress water deficit tolerance derived from *Solanum galapagense* accession LA1141 into cultivated tomato

**DOI:** 10.3389/fpls.2022.947538

**Published:** 2022-07-28

**Authors:** Sean Fenstemaker, Jin Cho, Jack E. McCoy, Kristin L. Mercer, David M. Francis

**Affiliations:** ^1^Department of Horticulture and Crop Science, The Ohio State University, Wooster, OH, United States; ^2^Department of Horticulture and Crop Science, The Ohio State University, Columbus, OH, United States

**Keywords:** thermal images, genomic selection (GS), proximal sensing, high-throughput phenotyping, inbred backcross method, canopy temperature (CT)

## Abstract

Crop wild relatives have been used as a source of genetic diversity for over one hundred years. The wild tomato relative *Solanum galapagense* accession LA1141 demonstrates the ability to tolerate deficit irrigation, making it a potential resource for crop improvement. Accessing traits from LA1141 through introgression may improve the response of cultivated tomatoes grown in water-limited environments. Canopy temperature is a proxy for physiological traits which are challenging to measure efficiently and may be related to water deficit tolerance. We optimized phenotypic evaluation based on variance partitioning and further show that objective phenotyping methods coupled with genomic prediction lead to gain under selection for water deficit tolerance. The objectives of this work were to improve phenotyping workflows for measuring canopy temperature, mapping quantitative trait loci (QTLs) from LA1141 that contribute to water deficit tolerance and comparing selection strategies. The phenotypic variance attributed to genetic causes for canopy temperature was higher when estimated from thermal images relative to estimates based on an infrared thermometer. Composite interval mapping using BC_2_S_3_ families, genotyped with single nucleotide polymorphisms, suggested that accession LA1141 contributed alleles that lower canopy temperature and increase plant turgor under water deficit. QTLs for lower canopy temperature were mapped to chromosomes 1 and 6 and explained between 6.6 and 9.5% of the total phenotypic variance. QTLs for higher leaf turgor were detected on chromosomes 5 and 7 and explained between 6.8 and 9.1% of the variance. We advanced tolerant BC_2_S_3_ families to the BC_2_S_5_ generation using selection indices based on phenotypic values and genomic estimated breeding values (GEBVs). Phenotypic, genomic, and combined selection strategies demonstrated gain under selection and improved performance compared to randomly advanced BC_2_S_5_ progenies. Leaf turgor, canopy temperature, stomatal conductance, and vapor pressure deficit (VPD) were evaluated and compared in BC_2_S_5_ progenies grown under deficit irrigation. Progenies co-selected for phenotypic values and GEBVs wilted less, had significantly lower canopy temperature, higher stomatal conductance, and lower VPD than randomly advanced lines. The fruit size of water deficit tolerant selections was small compared to the recurrent parent. However, lines with acceptable yield, canopy width, and quality parameters were recovered. These results suggest that we can create selection indices to improve water deficit tolerance in a recurrent parent background, and additional crossing and evaluation are warranted.

## Introduction

Approximately 1.2 billion people worldwide reside in areas with water scarcity, and this number is growing (Food and Agriculture Organization [FAO], 2021 accessed at: https://www.fao.org/land-water/water/drought/en/). In arid regions, population growth and economic development are exhausting renewable but finite water resources (Food and Agriculture Organization [FAO], 2021). Water deficit tolerance is imparted through morphological and physiological traits in plants. Traits used to indicate plant response to water deficit include, but are not limited to, leaf rolling, flower and fruit set, water use efficiency, recovery after re-watering, stomatal conductance, plant survival, leaf water potential, leaf osmotic potential, osmoregulation, transpiration rate, photosynthetic rate, enzymatic activities, pollen viability, and seed germination (Foolad, 2007; Galmés et al., 2011). Mechanical reduction of water loss can be imparted by changes in plant morphology or mechanisms that promote stomatal closure. Morphological traits, such as leaf size, shape, thickness, orientation, reflective capabilities, trichomes, and leaf angle, can modulate physiological response to deficit irrigation (Inoue et al., 1990; Kitaya et al., 1998). Additionally, water deficit stress may influence leaf expansion, osmotic adjustment, biomass partitioning, and stomatal characteristics (Koch et al., 2019; Pardo and VanBuren, 2021). Other tolerance mechanisms may include adjustments of carbon concentration and a reduction of photorespiration, leading to increased water use efficiency (Pardo and VanBuren, 2021). Morphological and physiological responses are often challenging to measure in studies with many treatments or large population sizes because there is a limited window of time to capture a response that is physiologically meaningful and genetically relevant (Smith et al., 2021).

Optimizing strategies to select for water stress tolerance may be a key to successfully exploiting genes from exotic germplasm. Plant canopy temperature has been proposed as a proxy to indicate stress under water deficit (Perera et al., 2020) and may provide an efficient phenotyping alternative to physiological measurements (Grossiord et al., 2020). Therefore, image-based phenotyping may provide an opportunity to increase the precision and throughput of trait measurement. For example, estimates of canopy temperature obtained from images could substitute as a measure of water deficit stress and is potentially a viable trait for the efficient assessment of plant physiological responses. Quantifying the genetic variation associated with specific traits offers a framework to identify and optimize appropriate phenotyping strategies. For plant breeding applications, improving the genetic resolution of trait measurements can increase the relative efficiency of selection procedures on a per cycle, per cost, and per time basis (Heffner et al., 2010).

The foundation of crop improvement is based on genetic variation underlying traits of interest. Previous studies have shown that wild tomato species possess tolerances to predatory insects, excessive moisture, salinity, and water deficit (Rick, 1973). The introgression of genes from wild tomato species that impart tolerance to abiotic stress specifically has been reported from *Solanum pimpinellifolium, Solanum peruvianum, Solanum cheesmaniae, Solanum habrochaites, Solanum chmielewskii*, and *Solanum pennellii* (Rick, 1978; Saeed and Fatima, 2021). Introgressions from the wild relative *S. pennellii* have improved tomato response under water deficit (Dariva et al., 2020). Accessing traits from wild relatives by conventional breeding is challenging because of reproductive barriers and linkage drag (Swarup et al., 2021). A wild tomato species endemic to the Galápagos Islands, *Solanum galapagense* (Darwin et al., 2003), is a potential donor of abiotic stress tolerance (Brozynska et al., 2016). *S. galapagense* is adapted to harsh environments, including low moisture and saline coastal habitats (Rick, 1956; Rush and Epstein, 1976; Pailles et al., 2020). The relative merit of a wild accession as a source of tolerance can be determined by using the observations made by collectors in native habitats (Rick, 1973, 1978). *S. galapagense* accession LA1141 was found growing on the interior walls of a crater on the island of Santiago on the north-facing slope, which received average annual precipitation from 1991 to 2020 of 7.7 cm (World Bank Group, 2021, accessed at: climateknowledgeportal.worldbank.org). The habitat described in the collection notes suggested that LA1141 is adapted to an arid environment (Castro, 1968, accessed at: tgrc.ucdavis.edu/Data/Acc/dataframe.aspx?start=AccSearch.aspx&navstart=nav.html) and is plausibly a suitable candidate to improve cultivated tomatoes through introgression. Introgression of tolerance from wild accessions from the more closely related red-fruited tomato, such as *S. galapagense*, may have advantages, such as greater recombination and potentially less linkage drag, compared to crosses with a more distantly related, green-fruited wild tomato relative like *S. pennellii* (Hamlin et al., 2020).

The objectives of this study were to evaluate methods of measuring plant response to water deficit and compare selection strategies to identify germplasm with lower canopy temperature, higher turgidity, higher stomatal conductance, and lower vapor pressure deficit (VPD). Using an inbred backcross strategy (Kabelka et al., 2002; Robbins et al., 2009), we created tomato populations derived from the tolerant donor parent, *S. galapagense* accession LA1141 (Fenstemaker et al., 2022). This population was used to do the following: (1) Improve and compare phenotyping workflows for measuring canopy temperature based on high throughput image analysis to indicate water deficit tolerance. (2) Associate measures of water deficit stress with single nucleotide polymorphisms (SNPs) to describe the genetic basis of low canopy temperature and high turgidity under water deficit in LA1141. (3) Discover and introgress chromosomal regions contributing to canopy temperature and severity of wilt in regions of the genome derived from accession LA1141. (4) Evaluate phenotypic and genomic-based selection strategies to identify water deficit tolerant progenies and evaluate the recovery of horticultural traits from an elite commercial parent in tolerant selections. Results from this study can be directly used to enhance the introgression of water deficit tolerance from wild relatives of tomato.

## Materials and methods

### Plant materials, crosses, and growing conditions

An inbred backcross (IBC) population was created using *S. galapagense* S.C. Darwin and Peralta (formerly *Lycopersicon cheesmaniae* f. minor) (Hook. f) C.H.Mull.) accession LA1141, as a source of traits for tomato improvement, and *Solanum lycopersicum* L. (formerly *Lycopersicon esculentum* Mill) OH8245 (Berry et al., 1991), as the recurrent parent (Fenstemaker et al., 2022). The IBC population was derived from an initial hybridization of *S. galapagense* LA1141 as the female parent and OH8245 as the male. BC_1_ progenies were used as females for further crosses to OH8245. The C.M. Rick Tomato Genetic Resources Center, University of California, Davis, provided the seed for LA1141. During these studies, IBC selections were further inbred to BC_2_S_5_. Selected BC_2_S_5_ progenies were advanced based on BC_2_S_3_ phenotypic values, genomic estimated breeding values (GEBVs), and random selection.

Seedling care for greenhouse and field trials followed the same protocol. Seeds were sown in 288-cell trays with a cell volume of 13 mL. Greenhouse temperatures were set to 27°C during the day and 25°C at night with a 16 h photoperiod. Photosynthetically active radiation (PAR) was supplied by natural sunlight, 1,000-W metal-halide lamps (Multi-Vapor^®^ GE Lighting, East Cleveland, OH, United States), and 1,000-W high-pressure sodium lamps (Ultra Sun^®^ Sunlight Supply, Vancouver, WA, United States) with a target threshold of 250 m^–2^ s^–1^ for natural sunlight before initiating artificial lighting to maintain light levels. PAR values in the greenhouse ranged from 250 to 637 μmol m^–2^ s^–1^ with an average of 391.4 μmol m^–2^ s^–1^. Fertilization was applied using a 20-20-20 fertilizer (20% N, 20% P_2_O_5_, and 20% K_2_O) (Jack’s professional All-Purpose Fertilizer, JR Peters INC., Allentown, PA, United States) and delivered at a concentration of 200 mg L^–1^ twice per week. For greenhouse evaluations, plants with three to five expanded leaves were transferred to 3.7-L plastic containers, filled with PRO-MIX (Premier Horticulture, Quakerstown, PA, United States), and spaced 30 cm apart on a greenhouse bench. Transplants in 3.7-L plastic containers were automatically irrigated four times per day, for 6 min, at a volume of 220 mL per irrigation cycle.

### Greenhouse evaluations of plants under water deficit

Greenhouse trials were conducted to evaluate water-deficit stress in the LA1141 and OH8245 parents, the segregating LA1141 × OH8245 BC_2_S_3_ families, and selections were advanced to the BC_2_S_5_ generation using phenotypic, genomic, and combined selection strategies or randomly chosen BC_2_S_3_ families advanced to the BC_2_S_5_ generation. The LA1141 and OH8245 parents were evaluated using a randomized complete block design with three blocks and three replicates within each block. The segregating BC_2_S_3_ families were evaluated in augmented designs as single replicates, with 36 replicates of each LA1141 and OH8245 parent distributed in a grid-like pattern corresponding to the row and column blocks across environmental gradients established between cooling pads, fans, and differences in solar radiation. The LA1141 and OH8245 parents were both used as over-replicated controls to correct for environmental variation in the greenhouse (Federer and Raghavarao, 1975; Bojacá et al., 2009). The BC_2_S_3_ families were evaluated in the greenhouse twice, in a summer environment and a fall environment. The BC_2_S_5_ advanced lines selected randomly or using phenotypic, genomic, and combined strategies were evaluated in an augmented design with three blocks, and a single replicate within each block. In the BC_2_S_5_ evaluation, each LA1141 and OH8245 parent was replicated nine times in each unique row by column spatial designation to account for environmental variation in the greenhouse as described above.

Water deficit treatments followed the same protocol in all greenhouse evaluations. When the plants reached the growth stage of six to eight expanded leaves in 3.7-L containers, irrigation was simultaneously withheld on all genetic treatments, and plants were evaluated daily, and the first evaluation happened at saturation. Pots were weighed at saturation, after 72 h of deficit irrigation, and at 144 h to estimate evapotranspiration. A Decagon GS3 sensor (Decagon Devices, Pullman, WA, United States) was calibrated for soilless potting media and used to measure volumetric water content (VWC) at saturation. The Decagon GS3 sensor utilized capacitance probes that measure real dielectric permittivity values and are generally preferred for water deficit evaluations compared to instruments that use time-domain reflectometry to measure soil water content changes in greenhouse pot experiments (Sharma et al., 2017). Estimates of VWC and gravimetric evaluations confirmed that starting saturation was even and consistent at the beginning of each experiment.

The leaf turgor status of individual genetic treatments was assessed daily during the dry-down phase on a whole plant basis using a visual turgor index as an estimation of whole plant wilt severity. Turgor index ratings ranged from 1 to 5 (5 = turgid, 4 = soft to the touch, 3 = beginning to wilt, 2 = wilted with complete loss of turgor, and 1 = dead), consistent with previous studies (Waterland et al., 2010). The turgor index was used to rate whole plant wilt status with higher scores indicating higher turgidity. Canopy temperature was measured with a handheld infrared thermometer (IRT) (Zhuhai JiDa Huapu Instrument Co., Hong Kong) and with an image-based methodology (see section High-throughoutput thermal image analysis). Canopy temperature was estimated with IRT by measuring the surface temperature (°C) of two fully expanded leaves per plant. IRT-estimated canopy temperature was monitored daily under ambient environmental conditions in the greenhouse between 10:00 and 12:00. The IRT was calibrated against a standard laboratory thermometer using a water bath calibration method (Horwitz, 1999). Additionally, the IRT was set to a constant emissivity of 0.97 for measurements of plant canopy consistent with previous studies (Kustas et al., 2012). Image-based whole plant canopy temperature was estimated using a FLIRONE GEN3 iOS thermal camera (FLIR Systems Wilsonville, OR, United States) and calibrated relative to water baths at a known temperature (Horwitz, 1999). The thermal camera was also set to a constant emissivity of 0.97. Images were captured against a standardized background using a 50 cm × 76 cm black polystyrene core foam board (Elmer’s Westerville, OH, United States) at 1 m; extraction of temperature data from images is further detailed in section “High-throughput thermal image analysis.” The IRT measurements and thermal images were recorded simultaneously.

The physiological response of LA1141, OH8245, and BC_2_S_5_ advanced selections were assessed daily during the dry-down phase and measured on a single, upper, and fully expanded leaf using the LI-600 porometer/fluorometer (LI-COR Biosciences, Lincoln, NE, United States). Measurements of stomatal conductance (g_*sw*_), vapor pressure deficit (VPD), and light-adapted chlorophyll fluorescence (PhiPS2) were taken under ambient conditions in the greenhouse between 10:00 and 12:00.

Phenotypic data collected on LA1141 and OH8245 plants grown under water deficit were analyzed as a fixed-effects model using the function “lm” in the R core package version 4.0.3 (R Core Team, 2020). The model *Y*_*ij*_ = μ + *genotype*_*i*_ + *Block*_*j*_ + *ε_*ij*_*: where *Y*_*ij*_ was the response variable, μ was the mean response of the parents, *genotype*_*i*_ represented the replicated LA1141 donor parent (*N* = 9) and OH8245 recurrent parent (*N* = 9), *Block*_*j*_ was used to estimate variation in the greenhouse across the air movement gradient established between cooling pads and fans, and *ε_*ij*_* was the associated experimental error. Factors with significant *p*-values (*p* < 0.05) were analyzed using Tukey’s Honest Significant Difference test, with the “HSD.test” function in the R package Agricolae (De Mendiburu, 2017).

The random-effects model, *Y_*ij*_* = μ + *genotype*_*i*_ + *Row*_*ij*_ + *Column*_*ij*_ + *ε_*ij*,_* was used to evaluate the BC_2_S_3_ families and over-replicated parental controls. The analysis was conducted using the “lmer” function in the R package lme4 (Bates et al., 2015). For this analysis, *Y*_*ij*_ was the response variable, *genotype*_*i*_ represented 160 individuals from the population as single replicates, and 36 replicates each of OH8245 and LA1141 as over-replicated controls. The environmental terms *Row*_*ij*_ and *Column*_*ij*_ were used to capture spatial variation within the greenhouse as described above, and *ε_*ij*_* was the associated error. Data were analyzed in the R core package version 4.0.3 (R Core Team, 2020). The significance of the main effects in these tests was determined by comparing a fully parameterized model to a model with a single term dropped using a likelihood ratio test based on a chi-square distribution (Snijdgers and Bosker, 2012). A significant *p*-value was interpreted as evidence that the parameter dropped was important to the fit of the model, and Bayesian Information Content (BIC) values were used to confirm that the full model provided a better fit to confirm the significance of genetic and environmental terms.

The germplasm screen of BC_2_S_3_ families was repeated twice, once in July and once in November. Best Linear Unbiased Predictors (BLUPs) were extracted for genetic treatments in the model using the “ranef” function in lme4 (Bates et al., 2015). BLUP values for canopy temperature and turgor response variables represented the spatially adjusted values for each BC_2_S_3_ family in each germplasm screen. The BLUPs estimated for canopy temperature and plant turgidity and were then averaged across the two experiments and used for the quantitative trait loci (QTL) mapping study and for developing phenotypic and genomic selection indices. Similarly, the random model described above was used to evaluate and analyze BC_2_S_5_ progenies in the selection strategies validation experiment. For this analysis, *Y*_*ij*_ was the response variable, *genotype*_*i*_ represented 30 individuals from the population replicated three times. The LA1141 and OH8245 parents were each replicated nine times. *Row*_*ij*_ and *Column*_*ij*_ were used to capture environmental variation as mentioned previously.

### High-throughput thermal image analysis

Thermal images were captured with a FLIRONE GEN3 iOS thermal camera as described above. Images were analyzed in a workflow that entailed extracting radiometric data as an integer matrix using the “readflirJPG” function in the R package Thermimage (Tattersall, 2021). The function “raw2temp” was used to convert the raw values obtained from the binary thermal image into estimated temperature with the equation (Eq. 1):


(1) temperature=PB/log(PR1/(PR2*(raw+PO))+PF)-273.15

Where *PB* was Planck’s constant B, the *log* was the base 10 logarithm, *PR1* was Planck’s constant R1, *PR2* was Planck’s constant R2, *raw* was a 16-bit encoded value associated with the radiance hitting the thermal camera sensor, *PO* was Planck’s constant O, *PF* was Planck’s constant F, and –273.15 was the conversion of temperature from K to °C. The Planck constant values were calibration constants that are specific to each FLIR thermal camera and were extracted using the software ExifTool (Harvey, 2020) as implemented in the R package “Thermimage” using the function “flirsettings (camvals = -*Planck*)”. The emissivity of the plant object, atmospheric temperature, and temperature of the ambient surroundings were extracted from the sensor on the FLIRONE GEN3 iOS thermal camera and were used to estimate plant canopy temperature.

Raw files with pixel values corresponding to the plant canopy surface temperature were then imported into the open-source software ImageJ (Java-based distribution Fiji) (Schindelin et al., 2012) for image processing and analysis. A collection of ImageJ functions and macros called “ThermImageJ” (Tattersall, 2019) was used to import data extracted from the thermal images, isolate regions of interest (ROI), and estimate plant canopy temperature. Histogram-based thresholding algorithms were accessed in the core ImageJ package using the steps “Image” > “Adjust” > “Threshold”. Image thresholding was performed to isolate the plant canopy ROI from the background of the image and employed the labeling operator *Tr* which labeled a pixel in the image only if its intensity *g* exceeded a specific threshold value *Tmin* (Eq. 2):


(2) Trα[g(x)≥Tmin]:g(x)→ 1(x)

Where 1 was the binary pixel label denoting the foreground value and *g* was the pixel intensity at the vector *x* (Prodanov and Verstreken, 2012). *Tmin* values can be determined using specific thresholding methods and we used “MEAN” described in Glasbey (1993). The “MEAN” method was chosen for our workflow because the proportion of total phenotypic variance attributed to genetic factors associated with canopy temperature was higher than the other tested thresholding methods (Supplementary Table 1). The “MEAN” thresholding algorithm was applied following these steps: “MEAN” > “Apply” > “set to NaN”, resulting in a 32-bit image with the plant canopy ROI isolated from the image background. We estimated canopy temperature from our final image following the steps: “Analyze” > “Measure”, which calculated the average estimated surface temperature of the plant canopy remaining in the image. Macros were written to automate and standardize the processes described above and to facilitate batch image analysis.

### Comparisons of phenotyping methods

Phenotypic measurements for selected BC_2_S_5_ progenies were compared using linear regression and variance partitioning to identify traits with higher genetic variance. The slope of the regression line was used to estimate the relationship of thermal image estimated canopy temperature to IRT, turgor ratings, and physiological traits measured with the LI-600. The random model described in section 3.2, above, was used to estimate the proportions of variance due to genetics (*genotype*_*i*_), environmental factors (*Row*_*ij*_ and *Column*_*ij*_), and experimental error (*ε_*ij*_*) in the models. The proportions of environmental and genetic variance provided an estimate of the broad-sense heritability and repeatability of each trait (Falconer and Mackay, 1996).

### Genotyping and linkage map construction

Genomic DNA was isolated from fresh, young leaf tissue from the 160 BC_2_S_3_ families and parental lines using a modified CTAB method (Fenstemaker et al., 2022). The 157 polymorphic SNP markers and linkage map construction were described previously (Fenstemaker et al., 2022). Briefly, a genetic linkage map was constructed using the R/qtl package version 1.47-9 in the R statistical software environment version 4.0.3 (Broman et al., 2003; Shannon et al., 2013; R Core Team, 2020) based on the LA1141 × OH8245 BC_2_S_3_ population (Fenstemaker et al., 2022). Marker data corresponding to the LA1141 × OH8245 BC_2_S_3_ was deposited in Zenodo.^1^ Map construction with the BC_2_S_3_ population was a compromise that provided genetic structure, captured a high percentage of elite parent background underlying important agronomic and horticultural traits, and offered opportunities to fix desirable donor alleles in further generations.

### Quantitative trait loci analysis in the LA1141 × OH8245 BC_2_S_3_ population

The QTL analysis was conducted with composite interval mapping (Zeng, 1994) using the “cim” function in the R/qtl package. Analysis was performed using a 2 centimorgan (cM) step, one marker selected as a cofactor, and a marker window set to 40 cm. The marker cofactor number and window size was due to limited recombination in the BC_2_S_3_ population. The Haley Knott regression (Haley and Knott, 1992) method was used for QTL detection. A significance threshold of LOD = 3.3 for both canopy temperature and plant turgor was determined by resampling the data (α = 0.05, *n* = 1,000; Churchill and Doerge, 1994). A cut-off of LOD = 2.4 significance threshold corresponded to *p* < 0.01. Genetic effects were estimated as differences between phenotypic averages expressed as regression coefficients using the “fitqtl” function with the argument “get.ests = TRUE” and “dropone = FALSE” in the R/qtl package. The percentage of phenotypic variance explained was estimated using the “fitqtl” function with the argument “dropone = TRUE” in the R/qtl package.

### Genomic selection models

Genomic estimated breeding values were calculated using ridge regression (RR) as implemented in the rrBLUP package in the R statistical software environment version 4.0.3 (Endelman, 2011; R Core Team, 2020). The RR computations were performed using the function “mixed.solve” in rrBLUP. Markers were considered as random effects associated with plant turgor and canopy temperature response variables. The estimated marker effects were used to calculate the GEBV of each LA1141 × OH8245 BC_2_S_3_ family. The equation used was: GEBV = X × MV: where GEBV was the vector of dimension (n, 1) containing the GEBVs for n families, X (n, m) was the matrix of scores for m markers and n families, and MV (m, 1) was a vector of marker effects for the m markers. GEBVs estimated from RR were used for genomic selection.

### Validation and comparison of selection methods

The phenotypic selection was based on the BC_2_S_3_ family visual ratings corresponding to plant turgor and canopy temperature values estimated using image-based methods. Phenotypic values were expressed as BLUPs estimated from the population screens described in section “High Throughput Phenotyping Using Thermal Images.” The BLUP values corresponding to each trait were sorted numerically and assigned a rank. The BC_2_S_3_ family with the highest value associated with plant turgor was ranked 1 (best), and the family with the lowest value associated with canopy temperature was ranked 1 (best). Plant turgor and canopy temperature BLUP ranks were summed into a single value that resulted in a rank-sum list. Top-ranking progenies (N = 10) were chosen according to this simplified multi-trait index (MTI). The selection intensity K was defined in standard deviation (SD) units relative to the mean. All phenotypic selections for plant turgor were made at a selection intensity of K = 1. Three canopy temperature phenotypic selections were made at a selection intensity of K = 2, and seven were made at a selection intensity of K = 1.

The genomic selection model mentioned above was used to calculate GEBVs for each trait. The GEBVs were sorted numerically and assigned a rank as described above. Canopy temperature GEBV and plant turgor GEBV ranks were summed into a single value used as an MTI as described in the section above, and top progenies were chosen based on this GS selection index. The top six selections for plant turgor GEBV were made at K = 2, with four additional selections at K = 1. All genomic selections for canopy temperature were made at K = 2. The final groups of selections consisted of phenotypic selections (Pheno, N = 9), genomic selections (GS, N = 8), randomly selected (Random, N = 10) families, and a group that was co-selected as top-ranking phenotypic and genomic selections (Pheno + GS, N = 3). These groups consisted of the top 10 ranking phenotypic selections, top 10 ranking genomic selections, and 10 randomly selected families totaling 30 progenies for further inbreeding.

The prediction abilities of the GS models were evaluated using cross-validation (theoretical accuracy) and empirical validation (realized accuracy). Cross-validation was conducted using a leave-one-out strategy (Liabeuf et al., 2018) on the BC_2_S_3_ families, which were considered our training population. Empirical validation was conducted using greenhouse performance data for plant turgor and canopy temperature measured on advanced BC_2_S_5_ lines derived from inbreeding selected BC_2_S_3_ families from the training population. The abilities of GS models to predict performance were estimated by two different models. First, the cross-validation prediction accuracy (r_*g*_) was evaluated using the Pearson coefficient of the correlations between GEBVs and phenotypic BLUPs in the BC_2_S_3_ families. Second, empirical validation r_*g*_ was evaluated using the Pearson coefficient of the correlations between the BC_2_S_3_ family GEBV and phenotypic BLUPs in the advanced BC_2_S_5_ selections. Additionally, the percentage of co-selection (% co-selection) was calculated using the number of selected families identified as both top 10 ranking phenotypic values at a minimum selection intensity of K = 1 and top 10 ranking GEBVs at a minimum selection intensity of K = 2 divided by the total number BC_2_S_3_ families selected using phenotypic values and GEBVs (N = 20).

The LA1141 × OH8245 BC_2_S_3_ phenotypic, genomic, and randomly selected families were advanced to the BC_2_S_5_ generation and evaluated in an augmented design in the greenhouse as described in section “Greenhouse evaluations of plants under water deficit.”

Trait BLUP values from selections were combined according to selection strategy, which was considered a treatment factor in the analysis. A fixed-effects model with selection strategy as a factor was used to determine if BC_2_S_5_ progenies chosen using selection strategies were significantly different from randomly advanced lines. The linear model used was *Y* ∼ μ + *Selection*+ ε: where *Y* was the trait BLUP, μ was the trial mean, *Selection* was the selection strategy used (GS, Phenotypic, Pheno + GS, and Random), and ε was experimental error. Factors with a significant *p*-value (*p* < 0.05) were analyzed using Tukey’s Honest Significant Difference with the “HSD.test” function in the R package Agricolae (De Mendiburu, 2017). The traits evaluated in the selection strategy validation experiment were turgor, canopy temperature, g_*sw*_, and VPD.

### Evaluation of selections for horticultural traits

Advanced genomic and phenotypic selections were also evaluated in a field trial to assess whether OH8245 horticultural traits were recovered. The field trial was designed as a randomized complete block design with two blocks and a single replicate in each block. The experimental unit was the plot. The field site was located at the Horticulture Unit 1 Research Farm in Wooster, Ohio. Each plot consisted of seven to ten plants and was spaced 30 cm apart in rows, with each row separated by 1.5 m. Maintenance of field plots followed standard practices for tomato production in the Midwest (Philips et al., 2021 accessed at: mdc.itap.purdue.edu). The genetic treatments consisted of the OH8245 recurrent parent, the BC_2_S_5_ phenotypic selections (N = 10), and BC_2_S_5_ genomic selections (N = 10). Seedlings were transplanted to the field four weeks after emergence. Plots were harvested when 80% of fruit in a plot reached the red ripe stage of maturity, which averaged 107 Julian calendar days. Before harvest, the plant canopy’s width and height were measured in cm by hand. Three plants were hand-harvested from the middle of each plot. The fruit was sorted into ripe, green, and cull maturity categories, and each group was weighed separately. Cull fruits were fruit with cracks, blemishes, or disease. Total yield was measured as the combined harvested weight of the three groups. A sub-sample of 20 fruit was analyzed using color and chemical traits associated with tomato fruit quality. Fruit color was measured on a cross-section of 11 fruit and shoulder cuts of nine fruit using the image-based software Tomato Analyzer (Darrigues et al., 2008). Soluble solids (Brix°) were quantified by filtering juice through a Kimwipe™ (Kimberly-Clark Corp., Neenah, WI, United States) and measured using a handheld refractometer (PAL-1, Atago U.S.A., Bellvue, WA, United States).

Field performance of OH8245 and BC_2_S_5_ advanced lines that were chosen using phenotypic and genomic selection was evaluated using the fixed effects model *Y_*ij*_* = μ + *genotype*_*i*_ + *Block*_*j*_+ *ε_*ij*_*: where *Y*_*ij*_ was the response variable, *genotype*_*i*_ represented the BC_2_S_5_ selections (N = 20), and the OH8245 recurrent parent, *Block*_*j*_ was replication within the field, μ was the trial mean associated with the yield or quality parameters, and *ε_*ij*_* was the error. Genetic and environmental factors with a significant *p-value* (*p* < 0.05) were analyzed with Tukey’s Honest Significant Difference using the “HSD.test” function in the R package Agricolae (De Mendiburu, 2017).

## Results

### LA1141 under water deficit stress

**FIGURE 1:**
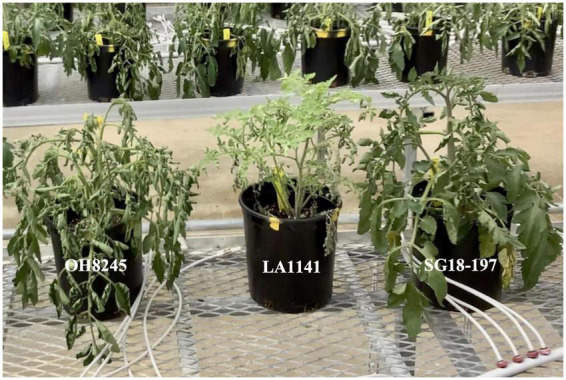
Response of, *Solanum lycopersicum* OH8245, *S. galapagense* accession LA1141 and LA1141 × OH8245 inbred backcross line SG18-197 at 72 h of water deficit. Turgor ratings ranged from 1 to 5 (5 = turgid, 4 = soft to the touch, 3 = beginning to wilt, 2 = wilted with complete loss of turgor, and 1 = dead), consistent with previous studies (Waterland et al., 2010). Accession LA1141 (labeled) **(center)** received a rating of 5, OH8245 **(left)** received a rating of 3, and LA1141 × OH8245 inbred backcross line SG18-197 **(right)** received a rating of 4.

**FIGURE 2:**
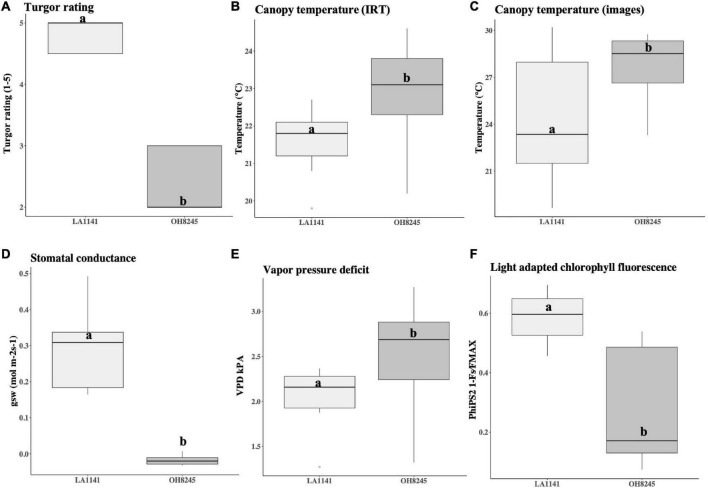
Boxplots comparing water-deficit stress response of the *Solanum galapagense* accession LA1141 donor parent (*N* = 9) to the *S. lycopersicum* OH8245 recurrent parent (*N* = 9). The traits measured were **(A)** turgor ratings ranging from 1 to 5 (5 = turgid, 4 = soft to the touch, 3 = beginning to wilt, 2 = wilted with complete loss of turgor, and 1 = dead) consistent with previous (Waterland et al., 2010). **(B)** Canopy temperature estimated using a handheld infrared thermometer (IRT) (Zhuhai JiDa Huapu Instrument Co., Hong Kong), and **(C)** from images captured with the FLIRONE GEN3 iOS thermal camera (FLIR Systems Wilsonville, OR, United States). Physiological measurements were taken with the LI-600 Porometer/ Fluorometer (LI-COR Biosciences, Lincoln, NE, United States) and included **(D)** stomatal conductance (g_*sw*_ mol m^–2^ s^–1^)**, (E)** vapor pressure deficit (VPD kPa), and **(F)** light-adapted chlorophyll fluorescence (PhiPS2 1-Fs/FMAX). Values are reported at the time point where they reach their maximums. Different letters indicate statistically significant differences among groups (Tukey’s Honest Significant Difference, *p* < 0.05).

Based on plant turgor, canopy temperature, and physiological measurements, LA1141 is more tolerant to water deficit stress than the OH8245 recurrent parent (Figures 1, 2 and Supplementary Table 2). Experiments were conducted over 144 h of deficit irrigation, with differences in turgor and canopy temperature observed between parents at 48 h through termination of the experiment (Supplementary Table 2). For simplicity, turgor and canopy temperatures are reported when they reach their maximums. Significant differences in turgor between LA1141 and OH8245 (*p* = 1.50e-15) are shown at 72 h (Figures 1, 2A and Supplementary Table 2). Accession LA1141 maintained a lower canopy temperature as measured by both an infrared thermometer (IRT) (*p* = 0.032) and thermal images (*p* = 0.049) (Figures 2B,C and Supplementary Table 2). Accession LA1141 and OH8245 exhibit different physiological responses, which become significant at 72 h of deficit irrigation (Supplementary Table 2). LA1141 maintains higher stomatal conductance (g_*sw*_) (*p* = 1.80e-07), lower vapor pressure deficit (VPD) (*p* = 0.025), and higher light-adapted chlorophyll fluorescence (PhiPS2) (*p* = 0.009) compared to OH8245 (Figures 2D–F and Supplementary Table 2). Observable and measurable differences in response between accession LA1141, OH8245, and their progenies (Figure 1) provided the basis for genetic studies describing water deficit tolerance derived from *S. galapagense.*

### High-throughput phenotyping using thermal images

**FIGURE 3:**
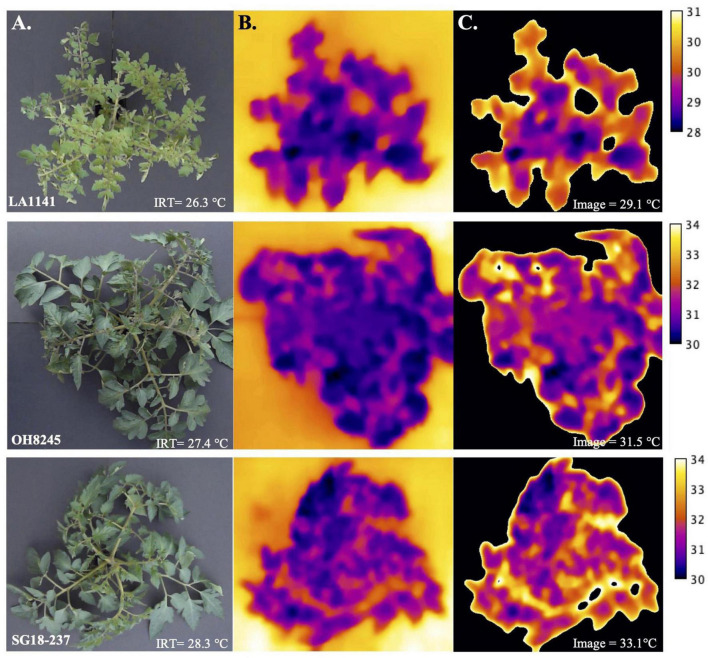
Estimating canopy temperature (°C) using images and an infrared thermometer (IRT) as a measure of plant water deficit stress. **(A)** Original images of LA1141 (top), OH8245 (middle), and inbred backcross line SG18-237 (bottom). The original image of the plant canopy was captured with the FLIRONE GEN3 iOS thermal camera (FLIR Systems Wilsonville, OR) and temperature (°C) was measured using a handheld IRT (Zhuhai JiDa Huapu Instrument Co., Hong Kong) simultaneously. **(B)** Plots of the temperature data extracted from thermal images using the function “plotTherm” in the R package Thermimage (Tattersall, 2021). **(C)** Histogram-based thresholding using “MEAN” (Glasbey, 1993) in the JAVA-based distribution of ImageJ “Fiji” (Schindelin et al., 2012). Images were analyzed in a workflow that entailed extracting radiometric data using the “readflirJPG” function in the R package Thermimage (Tattersall, 2021). Raw files with pixel values corresponding to the plant canopy surface temperature were then imported into the open-source software ImageJ (Schindelin et al., 2012) for image processing and analysis. A collection of ImageJ functions and macros called “ThermImageJ” (Tattersall, 2019) was used to import the temperature data extracted from the thermal images, isolate regions of interest (ROI), and estimate temperature. The image calibration bar was added to each individual image in panel C using the ImageJ macro “ThermImageJ” (Tattersall, 2019).

Thermal image-based phenotyping and analysis detected greater differences in canopy temperatures between genotypes under water deficit compared to canopy temperature measured with the infrared thermometer (IRT) (Figure 3). To test whether the high-throughput thermal image analysis pipeline offered advantages over the IRT, we evaluated LA1141, OH8245, and LA1141 × OH8245 BC_2_S_3_ families and BC_2_S_5_ selections for canopy temperature using both phenotyping approaches. Regression of canopy temperature measured with the IRT to values estimated by thermal images in the BC_2_S_3_ families showed a significant linear relationship (*p* < 2.20e-16, R^2^ = 0.30) (Supplementary Figure 3). Variance components for “Genotype” and experimental factors attributed to the environment, including “Row,” “Column,” and “error,” were partitioned to estimate the proportion of genetic variance associated with canopy temperature (Table 1). The total phenotypic variation partitioned into genetic effects associated with canopy temperature measured with IRT was 19.23% (Table 1). In contrast, the percentage of total phenotypic variance attributed to genetic factors measured with thermal images was 22.16% (Table 1). Thermal image estimated canopy temperature, therefore, provides higher repeatability.

**TABLE 1:** Percentage of total variance estimates for turgor ratings, canopy temperature, and LI-600 physiological measurements in *S. galapagense* LA1141, *S. lycopersicum* OH8245, and LA1141 × OH8245 BC_2_S_5_ progenies.

Sources of variation^a^	Turgor^b^	Image (°C)^c^	IRT (°C)^d^	g_*sw*_ mol m^–2^s^–1e^	VPD kPa^f^
Genotype	45.55	22.16	19.23	16.99	10.87
Row	11.40	0.00	0.04	2.59	2.19
Column	5.64	2.80	25.56	17.99	12.37
Residual	37.41	75.04	55.17	62.43	74.57

^a^Genotype is represented by *S. galapagense* accession LA1141 (N=9) and *S. lycopersicum* OH8245 (N=9), and advanced inbred progenies that have been backcrossed to OH8245 two times and self-pollinated five times (BC_2_S_5_) (N = 30, replicated three times). Row and Column were used as environmental terms to capture spatial variation across the greenhouse and each row by column location contained both replicated parental controls.

^b^Plant turgor ratings ranged from 1 to 5 (5 = turgid, 4 = soft to the touch, 3 = beginning to wilt, 2 = wilted with complete loss of turgor, and 1 = dead) consistent with previous studies (Waterland et al., 2010).

^c^Whole plant canopy temperature (°C) measured using a FLIRONE GEN3 iOS thermal camera (FLIR Systems Wilsonville, OR, United States).

^d^Leaf surface temperatures (°C) of two fully expanded leaves per plant measured with infrared thermometer (Zhuhai JiDa Huapu Instrument Co., Hong Kong).

^e^Stomatal conductance to H2O (mol m^–2^s^–1^) measured with the LI-600 porometer/fluorometer (LI-COR Bioscienes, Lincoln, NE, United States).

^f^Vapor pressure deficit kPa at leaf temperature measured with the LI-600 porometer/fluorometer (LI-COR Bioscienes, Lincoln, NE, United States).

Comparisons between image-based canopy temperature at 48 h and turgor ratings at 72 h show a significant correlation (*p* = 0.031), suggesting that canopy temperature can predict the onset of wilt (Supplementary Figure 4A). Image-based canopy temperature measurements at 48 h are also significantly correlated to g_*sw*_ (*p* = 0.023) and VPD (*p* = 0.002), but not to PhiPS2 (*p* = 0.583) at 72 h, suggesting that canopy temperature is predictive of g_*sw*_ and VPD (Supplementary Figures 4B–D). Notably, a higher percentage of phenotypic variance is partitioned into genetic effects associated with canopy temperature than g_*sw*_, VPD, and PhiPS2 (Table 1). However, the percentage of phenotypic variance partitioned into genetic effects associated with turgor is higher than canopy temperature and physiological measurements (Table 1). Still, an image-based canopy temperature appears to be a suitable proxy for more intensive physiological measurements, such as g_*sw*_ and VPD in our water deficit germplasm screens. Finally, the ability of images to predict the onset of wilt demonstrates that image-based measurements can improve the efficiency of germplasm screens.

### LA1141 × OH8245 BC_2_S_3_ families under water deficit stress

The BC_2_S_3_ families that differed from the trial mean by a selection intensity of K = 1 indicated tolerance or susceptibility to water deficit stress. Tolerance in specific LA1141 × OH8245 BC_2_S_3_ families for canopy temperature and turgor was recovered. Germplasm evaluations were conducted in the greenhouse in two seasonal environments (July and November). A summary of greenhouse conditions in summer and fall environments is provided (Supplementary Table 5). Genetic effects for turgor (*p* < 2.20e-16) and canopy temperature (*p* = 8.17e-07) were significantly different (Supplementary Table 6). Additionally, summer and fall environments were significantly different for turgor (*p* < 2.20e-16) but not significantly different for canopy temperature (*p* = 0.736) (Supplementary Table 6). The environmental term “Column” corresponds to a solar radiation gradient in these experiments. The term “Row” corresponds to an air movement gradient between the greenhouse cooling pad and fans. The interaction between the seasonal environment and row, or column, terms represent a unique greenhouse position within each environment. The “Environment × Row” term was significantly different for turgor (*p* = 0.007) but not canopy temperature (*p* = 0.371) (Supplementary Table 6). However, Environment × Column was significantly different for both turgor (*p* = .0002) and canopy temperature (*p* = 5.05e-14) (Supplementary Table 6). The experimental design used over-replicated checks to estimate the best linear unbiased predictors (BLUPs) as described in the methods section “Greenhouse evaluations of plants under water deficit” to account for the variation in the greenhouse described above. Trait values expressed as BLUPs exhibit shrinkage around the mean and provided conservative estimates of turgor and canopy temperature adjusted to environmental differences based on the over-replicated LA1141 and OH8245 parental checks.

Additionally, estimates of evapotranspiration were not significantly different based on genotype (*p* = 0.461) but were significantly different between experimental environments (*p* = 3.42e-08) (Supplementary Table 6). However, the environmental interaction factors Environment × Row (*p* = 0.089) and Environment × Column (*p* = 0.732) were not significantly different (Supplementary Table 6). These results suggested that the position in the greenhouse and genetic differences for estimated evapotranspiration within an experimental environment did not explain significant differences in water-deficit stress response. Differences detected between the screening environments are not surprising because the average temperature in the greenhouse was higher in the summer compared to the fall (Supplementary Table 5). Consequently, observed genetic variation in the germplasm appears to be independent of estimated evapotranspiration, both for canopy temperature and plant turgor. These traits were subsequently used in interval mapping studies, genomic selection models, and the development selection indices.

### Quantitative trait loci analysis in the LA1141 × OH8245 BC_2_S_3_ population

**FIGURE 4:**
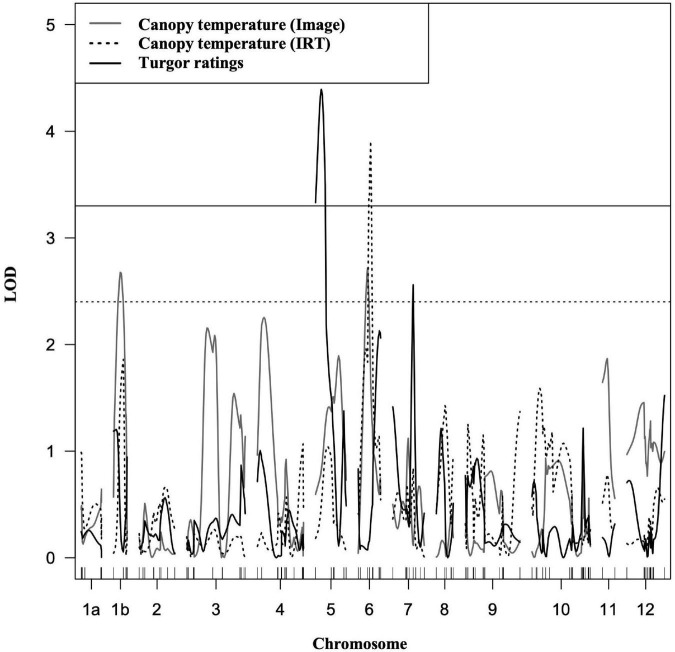
Composite interval mapping of LA1141-derived tolerance to water deficit stress. The traits measured were canopy temperature estimated using images (**grey**), canopy temperature estimated with an infrared thermometer (dotted), and plant turgor ratings (black) ranging from 1 to 5 (5 = turgid, 4 = soft to the touch, 3 = beginning to wilt, 2 = wilted with complete loss of turgor, and 1 = dead) consistent with previous studies (Waterland et al., 2010). Traits were measured in the LA1141 × OH8245 BC_2_S_3_ inbred backcross families (N = 160). The y-axis is the logarithm of the odds (LOD). The solid (black) horizontal line (LOD = 3.3) is the resampled LOD significance cutoff (α = 0.05, N = 1,000 permutations). The dotted (gray) horizontal line (LOD = 2.4) represents a significance level of *p* < 0.01. The x-axis represents linkage groups corresponding to the 12 chromosomes in tomato and chromosome distance in centimorgans (cM). The genetic distance was calculated using the Kosambi function Kosambi (1943) to correct for multiple crossovers.

Four putative QTLs were identified, and explained between 6.6 and 9.49% of the phenotypic variation for canopy temperature and turgor (Figure 4 and Table 2). Phenotypic values were expressed as an average of trait BLUPs across environments with a mean of 0, and the effect of an allele substitution was expressed relative to the mean. All QTLs contributing to water deficit tolerance were derived from the LA1141 donor parent (Table 2). A region on the distal arm of chromosome 1 (linkage group 1b) had a LOD score of 2.66, explained 6.6 % of the total phenotypic variation, and lowered canopy temperature by 0.02°C (Table 2). A region on the proximal arm of chromosome 6 had a LOD score of 3.46, explained between 6.02 and 9.09 % of the total phenotypic variation, and lowered canopy temperature by 0.03°C (Table 2). A region on the proximal arm of chromosome 5 had a LOD score of 3.33, explained 9.14 % of the total phenotypic variation, and increased turgor ratings between 0.33 and 0.36 units (Table 2). A region on the distal arm of chromosome 7 had a LOD score of 2.5, explained 6.78% of the phenotypic variation, and increased ratings associated with higher turgor by 0.42 units (Table 2). The QTL found on chromosomes 1, 5, and 7 were detected in both individual screens and the combined dataset (Table 2). The QTL associated with canopy temperature detected on chromosome 6 was detected in the summer and combined environments but not the fall environment (Table 2).

**TABLE 2:** Quantitative trait loci (QTL) and flanking single nucleotide polymorphism (SNP) markers associated with canopy temperature and turgor ratings in *S. galapagense* LA1141 x *S. lycopersicum* OH8245 BC_2_S_3_ families.

	LA1141 X OH8245 BC_2_S_3_									
Trait^a^	SNP marker	LOD^b^	*p* ^c^	QTL × seasonal environments^d^	Donor allele	Allele substitution effect^e^	Percent phenotypic variance explained^f^	Chromosome	Physical position (bp)^g^	Genetic position (cM)^h^
Thermal image canopy temperature	solcap_snp_sl_2234	0.00	0.998	Summer, fall, combined	LA1141	0.00	0.001	1b	79025804	00.00
	solcap_snp_sl_14323	2.66	0.004	Summer, fall, combined	LA1141	–0.02	6.60	1b	87223580	20.37
	solcap_snp_sl_13404	1.41	0.041	Summer, fall, combined	LA1141	–0.02	3.98	1b	88561836	25.81
	solcap_snp_sl_14458	2.71	0.002	Summer, combined	LA1141	–0.03	7.18	6	36520866	19.42
	solcap_snp_sl_1337	2.19	0.007	Summer, combined	LA1141	–0.03	6.02	6	37305722	23.28
	solcap_snp_sl_12757	1.00	0.090	Summer, combined	LA1141	–0.01	2.8	6	38186675	29.74

IRT canopy temperature	solcap_snp_sl_14458	1.97	0.010	Summer, combined	LA1141	–0.02	5.53	6	36520866	19.42
	solcap_snp_sl_1337	3.46	0.000	Summer, combined	LA1141	–0.03	9.49	6	37305722	23.28
	solcap_snp_sl_12757	2.15	0.007	Summer, combined	LA1141	–0.03	6.01	6	38186675	29.74

Turgor ratings	solcap_snp_sl_19102	3.33	0.000	Summer, fall, combined	LA1141	0.33	9.14	5	1909149	00.00
	solcap_snp_sl_5050	1.84	0.015	Summer, fall, combined	LA1141	0.36	5.15	5	6045160	32.00
	solcap_snp_sl_22065	0.26	0.54	Summer, fall, combined	LA1141	0.07	0.76	7	3718124	34.18
	solcap_snp_sl_5861	2.5	0.003	Summer, fall, combined	LA1141	0.42	6.78	7	59688274	42.51
	solcap_snp_sl_7025	0.104	0.29	Summer, fall, combined	LA1141	0.08	0.97	7	63561726	57.45

^a^Tolerance to water deficit measured as canopy temperature and plant turgor ratings in the OH8245 × LA1141 families that were backcrossed twice to OH8245 and self-pollinated three times (BC_2_S_3_). Thermal image canopy temperature and Infrared thermometer (IRT) both represent maximum canopy temperature values. Trait values were expressed as Best Linear Unbiased Predictors (BLUPs).

^b^Logarithm to base 10 (LOD) scores. A significance threshold of LOD = 3.3 for both canopy temperature and plant turgor was determined by resampling the data (α = 0.05, *n* = 1,000; Churchill and Doerge, 1994). A cut-off of LOD = 2.4 significance threshold corresponded to *p* < 0.01.

^c^The *p-value* retrieved using the Haley-Knott regression formula: y ˜ Q1, where y is the response variable and Q1 is the marker.

^d^Significant (*p*=<.01) QTLs detected in summer environments, fall environments, and in the combined dataset.

^e^Genetic effects evaluated as differences between phenotype averages expressed as regression coefficients.

^f^Percent variance explained estimated by 1 – 10^–2^ ^*LOD/n*^, where n is the sample size and LOD is the LOD score for the marker.

^g^Physical position in base pairs corresponds to the Tomato Genome version SL4.0 (Hosmani et al., 2019).

^h^Genetic position corresponds to the LA1141 x OH8245 BC_2_S_3_ linkage map previously developed (Fenstemaker et al., 2021).

### Validation of selection strategies

Prediction accuracy (r_*g*_) was evaluated with cross validation in the LA1141 × OH8245 BC_2_S_3_ training population and empirically in BC_2_S_5_ lines derived from further inbreeding. Cross-validation correlations between GEBVs and phenotype were significant for canopy temperature (*p* = 1.53e-15), and accuracy was r_*g*_ = 0.57. Similarly, correlations for turgor were significant (*p* = 4.61e-09) with an accuracy of r_*g*_ = 0.44 (Table 3). Correlations between GEBVs based on the BC_2_S_3_ training population and observed values of BC_2_S_5_ lines were significant for canopy temperature (*p* = 0.009, r_*g*_ = 0.57) and turgor (*p* = 0.021 r_*g*_ = 0.31) (Table 3).

**TABLE 3:** Evaluation of accuracy and genetic gain for selection strategies during inbreeding of the *S. galapagense* LA1141 × *S. lycopersicum* OH8245 population.

Population^a^		Canopy temperature °C^b^	Turgor ratings^c^
BC_2_S_3_ families	Minimum	18.50	1
	Maximum	28.90	5.00
	Mean	24.50	3.14
	s.d.^d^	1.56	1.18
	**Cross validation (r_*g*_)** ^e^	**0.57 (*p* = 1.53e-15)**	**0.44 (*p* = 4.61e-09)**
BC2S5 families	Minimum	18.05	2.16
	Maximum	27.74	4.07
	Mean	22.13	3.05
	s.d.	2.44	0.53
	**Empirical validation (r_*g*_)^f^**	**0.47 (*p* = 0.009)**	**0.31 (*p* = 0.021)**
BC_2_S_5_ GS	Minimum	22.01	2.61
	Maximum	25.97	3.49
	Mean	23.71	3.15
	s.d.	1.51	0.38
	**Genetic gain^g^**	**-0.79**	**0.01**
BC_2_S_5_ Pheno + GS	Minimum	20.66	2.76
	Maximum	25.37	3.93
	Mean	23.07	3.44
	s.d.	2.35	0.61
	**Genetic gain**	**-1.43**	**0.31**
BC_2_S_5_ Phenotype	Minimum	22.38	2.76
	Maximum	25.06	4.08
	Mean	23.93	3.41
	s.d.	0.86	0.47
	**Genetic gain**	**-0.57**	**0.27**
BC_2_S_5_ Random	Minimum	22.41	2.17
	Maximum	30.05	3.35
	Mean	26.81	2.64
	s.d.	2.87	0.39
	**Genetic gain**	**2.31**	**-0.5**

^a^Population represents LA1141 × OH8245 were backcrossed twice to OH8245 and self-pollinated three tiems (BC_2_S_3_) (*N* = 160) and advanced selections that underwent additional self-pollination (BC_2_S_5_) based on genomic estimated breeding values (GEBVs) (GS, *N* = 8), LA1141 × OH8245 BC_2_S_3_ canopy temperature and turgor best linear unbiased predictors (BLUPs) (Pheno, *N* = 9), a combination of the two (GS + Pheno, *N* = 3), and randomly advanced lines (Random, *N* = 10).

^b^Canopy temperature measured as whole plant canopy temperature (°C) using a FLIRONE GEN3 iOS thermal camera (FLIR Systems Wilsonville, OR, United States).

^c^Plant turgor based on a rating scale ranged from 1 to 5 (5 = turgid, 4 = soft to the touch, 3 = beginning to wilt, 2 = wilted with complete loss of turgor, and 1=dead) consistent with previous studies (Waterland et al., 2010).

^d^Standard deviation.

^e^Correlation coefficient between genomic estimated breeding values (GEBVs) and phenotypic values in BC_2_S_3_ progenies (training population).

^f^Correlation coefficient between genomic estimated breeding values (GEBVs) and phenotypic values in the advanced BC_2_S_5_ progenies.

^g^Increase in performance through selection (lower canopy temperature and higher turgor scores). Bold values are self-evident.

**FIGURE 5:**
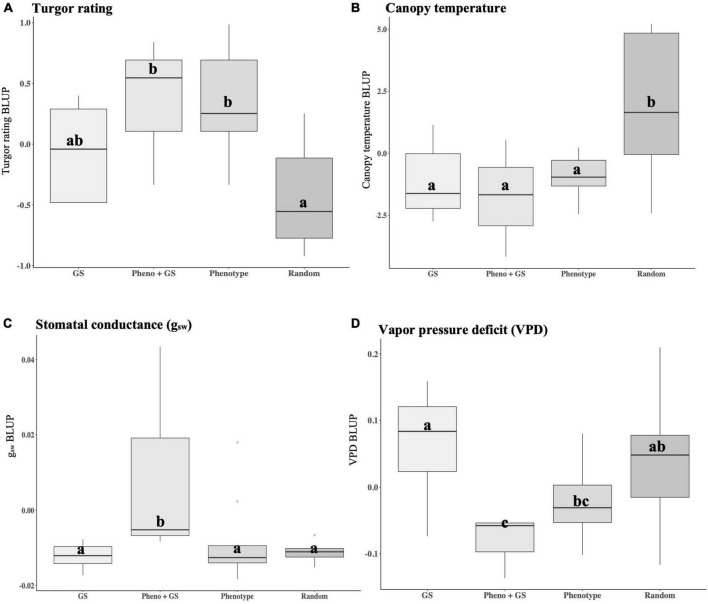
Boxplots comparing group performance of groups corresponding to selection strategies under water deficit. Traits were measured as best linear unbiased predictors (BLUPs) and include **(A)** plant turgor ratings, **(B)** canopy temperature, **(C)** stomatal conductance (g_*sw*_), and **(D)** vapor pressure deficit (VPD). Selection strategy groups were based on GEBVs (GS, *N* = 8), LA1141 × OH8245 BC_2_S_3_ canopy temperature, and turgor BLUPs (Pheno, *N* = 9), a combination of the two (GS + Pheno, *N* = 3), and randomly advanced lines (Random, *N* = 10). Each IBL was replicated three times within the selection strategy. Turgor ratings ranged from 1 to 5 (5 = turgid, 4 = soft to the touch, 3 = beginning to wilt, 2 = wilted with complete loss of turgor, and 1 = dead) as described previously (Waterland et al., 2010). Canopy temperature was estimated using the FLIRONE GEN3 iOS thermal camera (FLIR Systems Wilsonville, OR, United States). Physiological measurements were measured with the LI-600 Porometer/Fluorometer (LI-COR Biosciences, Lincoln, NE, United States) and included stomatal conductance (g_*sw*_ mol m^–2^ s^–1^) and vapor pressure deficit (VPD kPa). Values are reported at the time point where they reach their maximums. Different letters indicate statistically significant differences among groups (Tukey’s honestly significant difference, *p* < 0.05).

Improvement in plant performance in our experiments is demonstrated by high plant turgor, low canopy temperature, high g_*sw*_, and low VPD. Selection strategies based on phenotype, genomic selection models and a combination of the two were compared to randomly advanced lines. The selection strategy was significantly different from random selections for turgor (*p* = 0.004), canopy temperature (*p* = 0.006), g_*sw*_ (*p* = 0.026), and VPD (*p* = 0.046) (Supplementary Table 7). Phenotypic, genomic, and combined strategies resulted in positive gain under selection, with selected lines showing higher turgor and lower canopy temperatures under water deficit. Differences between the BC_2_S_3_ families and BC_2_S_5_ lines chosen based on GEBV resulted in gain under selection by increasing turgor by 0.01 units (Table 3). Lines selected using combined strategies resulted in gain under selection by increasing turgor ratings by 0.31 on the five-point scale (Table 3). Phenotypically selected BC_2_S_5_ advanced lines resulted in gain under the selection of 0.27 units (Table 3). In contrast, differences between the BC_2_S_3_ training population and the randomly advanced BC_2_S_5_ progenies did not result in gain under this selection and lowered turgor ratings by 0.50 on the five-point scale (Table 3). Importantly, phenotypic, and combined strategies had higher turgor compared to randomly advanced progenies (Figure 5A). However, chosen progenies using genomic selection alone did not have different turgor ratings when compared to phenotypic, combined, or random selection (Figure 5A).

Similarly, selection for lower canopy temperature resulted in observed genetic gains. Differences between the BC_2_S_3_ families and BC_2_S_5_ lines chosen based on GEBVs for canopy temperature lowered the canopy temperature to 0.79°C (Table 3). Lines selected using combined strategies lowered the canopy temperature to 1.43°C (Table 3). Phenotypically selected BC_2_S_5_ progenies resulted in gain under selection by lowering canopy temperature to 0.57°C (Table 3). In contrast, random selection raised canopy temperature by 2.31°C (Table 3). In conclusion, all selection strategies lowered canopy temperature compared to random selection (Figure 5B).

Selected water deficit tolerance based on high turgor and low canopy temperature also had the result of improving physiological measurements. Statistical differences for physiological measurements were observed for advanced lines chosen using combined selection strategies. Selected lines based on combined strategies maintained higher g_*sw*_ and lower VPD compared to randomly advanced lines (Figures 5C,D) suggesting that genomic selection can be used to augment phenotypic selection and indirectly select for improved plant physiological status under water deficit. Therefore, genomic, and phenotypic selection for turgor and canopy temperature are indirect methods to improve plant physiological response under water deficit stress. On average, genomic, phenotypic, and the combined strategies had g_*sw*_ values that were 0.007 mol H_2_O m^–2^ s^–1^ higher and VPD values that were 0.047 kPa lower relative to randomly advanced lines (Figure 5). These results suggested that progress toward tolerance to water deficit stress measured by turgor, canopy temperature, g_*sw*_, and VPD was achieved with phenotyping workflows and selection indices.

To gain additional insight into the genetics of low canopy temperature and high turgor under water deficit stress, we evaluated our selections for recovery of putative QTL derived from LA1141. Four selections based on phenotype (20% of total selections) possess the LA1141 introgression on chromosome 1b associated with canopy temperature (Table 2). First selection based on GEBV and second selection based on phenotype (15% of total selections) have the introgression from LA1141 on chromosome 6 associated with canopy temperature (Table 2). Three selections based on GEBV, four based on phenotype, and one selected with combined strategies (40% of total selections) have the introgressions from LA1141 on chromosome 5 associated with high turgor (Table 2). Three selections based on phenotype, one selected by combined strategies, and one selected based on genomic selection (20% of total selections) have the introgression from LA1141 on chromosome 7 associated with high turgor (Table 2). The expected frequency of a LA1141 allele in the BC_2_S_3_ population is 12.5%. The observed frequency of QTLs is, therefore, higher than expected on chromosome 1b (χ^2^ = 5.14, *p* = 0.022), chromosome 5 (χ^2^ = 69.14, *p* = 0.001), and chromosome 7 (χ^2^ = 5.14, *p* = 0.022). However, recovery of the QTL on chromosome 6 approaches the expected frequency (χ^2^ = 0.57, *p* = 0.450).

### Evaluation of BC_2_S_5_ selections for horticultural traits

A field trial was conducted to evaluate the performance of advanced BC_2_S_5_ selections that were chosen using genomic and phenotypic strategies to establish whether important agronomic and quality traits from the OH8245 recurrent parent were recovered. The fruit size of all BC_2_S_5_ selections was smaller (*p* = 8.07e-13) than the OH8245 (Table 4). However, some BC_2_S_5_ selections have acceptable yield, canopy width, fruit color, and fruit quality (Table 4). Acceptable values of traits were based on ranges observed in processing tomato germplasm (Merk et al., 2012). The acceptable range for canopy width in processing tomatoes is between 75 to 110 cm. In our trial, 65% of selections had an acceptable canopy size, while 25% had canopy size that was too small, and 10% had canopy sizes that were too large (Table 4). Lower values of hue, an angular measurement, represent the visible property of color. Values of L*, a coordinate that indicates the darkness (0) to lightness (100) of color, are associated with increased redness of tomatoes. Ten percent of our selections had improved L* values relative to the recurrent parent, and 100% of selections had an acceptable range of L* (Table 4). Additionally, 10% had improved hue measurements relative to the recurrent parent, and all selections had an acceptable range of hue (Table 4). Low estimates of the physiological color disorder, yellow shoulder (%YSD), are also associated with improved tomato color (Table 4). Forty-five percent of selections exceeded the acceptable cutoff for %YSD (Table 4).

**TABLE 4:** Evaluation of the LA1141 × OH8245 BC_2_S_5_ selected progenies for horticultural traits compared to the OH8245 recurrent parent.

	Field traits^y^					Color traits^x^		
Genotype^z^	Average fruit weight (g)	Total yield (t ha^–1^)	Marketable yield (t ha^–1^)	Canopy width (cm)	Brix°	% YSD	L*	Hue
OH8245	**68.9 a**	**111.5 a**	**53.5 abcd**	**86 abc**	**5.05 bcde**	**20.30 bc**	**42.81 ab**	**50.55 bc**
SG18-257	55.3 b	93.4 abcd	49.0 abcd	88.5 abc	5.45 abcde	18.12 bc	39.11 abc	50.19 bc
SG18-129	44.5 c	112.7 a	78.2 abc	85 bc	5.50 abcde	11.91 c	36.99 c	48.50 c
SG18-121	36.3 cd	102.7 abc	60.9 abcd	81.5 bc	4.80 bcde	25.66 bc	40.67 abc	52.39 bc
SG18-251	36.3 cd	92.4 abcd	53.2 abcd	67.5 c	4.80 bcde	14.91 c	40.10 abc	50.36 bc
SG18-195	34.5 de	91.1 abcd	61.5 abcd	73.5 bc	4.45 de	25.69 bc	42.27 abc	52.94 bc
SG18-197	33.6 def	69.8 abcd	25.5 bcd	127 ab	5.65 abcde	17.57 bc	40.78 abc	50.56 bc
SG18-223	29.9 defg	90.9 abcd	48.5 abcd	82.5 bc	4.85 bcde	39.39 abc	39.94 abc	54.35 abc
SG18-295	29.9 defg	56.5 bcd	18.8 d	102.5 abc	4.85 bcde	31.97 abc	38.76 abc	54.00 abc
SG18-165	29.0 defg	84.0 abcd	60.7 abcd	58.5 c	4.60 cde	40.22 abc	40.17 abc	54.83 abc
SG18-167	29.0 defg	52.7 cd	15.8 d	98 abc	6.15 abc	61.48 a	43.21 a	62.16 a
SG18-143	28.1 defg	83.1 abcd	43.8 abcd	77.5 bc	5.20 bcde	36.78 abc	39.16 abc	54.49 abc
SG18-177	25.4 efg	108.5 abc	89.6 a	108.5 abc	4.85 bcde	49.28 ab	41.81 abc	57.94 ab
SG18-292	24.5 fgh	102.2 abc	41.6 abcd	141 a	6.45 ab	38.73 abc	40.32 abc	55.74 abc
SG18-145	22.7 ghi	96.4 abcd	40.4 abcd	95 abc	7.00 a	14.71 c	37.24 bc	48.52 c
SG18-182	20.9 ghi	106.0 abc	79.4 ab	75 bc	4.75 cde	38.00 abc	41.26 abc	53.29 bc
SG18-188	15.4 hij	41.4 d	13.6 d	92 abc	5.80 abcde	31.30 abc	41.26 abc	56.17 abc
SG18-248	14.5 ij	43.2 d	19.3 bcd	79.5 bc	6.05 abcd	36.11 abc	39.15 abc	54.14 abc
SG18-255	10.0 j	65.2 abcd	38.9 abcd	71 c	4.20 e	42.07 abc	38.25 abc	54.83 abc
Mean	**31.0**	**84.4**	**46.9**	**88.94**	**5.28**	**31.27**	**40.17**	**53.47**
HSD (*p* < 0.05)^W^	**9.9**	**56.1**	**60.2**	**55.92**	**1.65**	**33.38**	**5.79**	**8.21**

^z^OH8245 is the *S. lycpersicum* elite parent. The remaining genotypes are selected LA1141 × OH8245 progenies that were backcrossed twice to OH8245 and self-pollinated five times (BC_2_S_5_). Phenotypic selections include SG18-129, SG18-143, SG18-177, SG18-195, SG18-197, SG18-248, SG18-251, and SG18-295. Genomic selections include SG18-121, SG18-145, SG18-167, SG18-182, SG18-188, SG18-255, SG18-257, and SG18-292. Combined selection strategies include SG18-165 and SG18-223.

^y^Average fruit weight (g) of 25 randomly sampled fruit divided by 25 to estimate average fruit weight. Harvested fruit was sorted into marketable, green, and cull groups and each group are weighed separately. Total yield was measured as harvested weight of the three groups.

^x^Color data measured with Tomato Analyzer (Darrigues et al., 2008) on a sub sample of 25 fruit from each plot. Yellow shoulder disorder (%YSD), represents yellow, green-yellow color.

L* coordinate indicates darkness (0) to lightness (100) of color. Hue is an angular measurement representing the visible property of color.

^w^The same letters in each treatment indicate non-significant differences among genotypes at *p* < 0.05 based on Tukey’s honest significant difference (HSD). Minimum significant difference is reported. Bold values are self-evident.

All selections had acceptable Brix° values relative to the OH8245 recurrent parent (Table 4). One selection had a higher Brix° than OH8245. However, this selection ranked 14th out of 20 in fruit size (Table 4). A BC_2_S_5_ selection chosen based on phenotype, SG18-129, and a BC_2_S_5_ selection chosen with genomic selection, SG18-145, had improved tomato color relative to OH8245. SG18-129 and SG18-145 also had acceptable canopy sizes (Table 4). Additionally, BC_2_S_5_ selection SG18-129 had numerically higher yield and numerically higher Brix° than OH8245. However, those differences in yield were not significant (Table 4).

## Discussion

### Response of LA1141 to water deficit stress

*Solanum galapagense* accession LA1141 and *S. lycopersium* OH8245 have different physiological responses to water deficit stress. Both morphological and physiological responses can contribute to water deficit tolerance (Koch et al., 2019; Pardo and VanBuren, 2021). Accession LA1141 demonstrates the ability to withstand deficit irrigation for as much as 144 h before wilting is observed. The physiological basis of this tolerance requires further investigation, but a mechanism mediated by stomatal conductance (g_*sw*_) is plausible. Similarities of g_*sw*_ at saturation and genetic variation in advanced selections are promising indicators that it is possible to select for g_*sw*_ while maintaining plant growth and yield. Previous studies have shown that VPD and g_*sw*_ are suitable proxies for osmotic adjustment and yield maintenance in plants grown under water deficit stress (Bazzer and Purcell, 2020). Accession LA1141 and advanced selections have lower VPD, higher g_*sw*_, and lower canopy temperature after 72 h of water deficit compared to OH8245 and randomly advanced lines, suggesting genetic variation for osmotic adjustments under water deficit stress is present in the population. The putative osmotic adjustment mechanism appears to be independent of leaf anatomy as advanced lines are 87.5% recurrent parent genome and most of the tolerant selections possess the recurrent parent leaf morphological phenotype. For example, the phenotypic selection SG18-197 is shown (Figure 1) and leaf morphology is characteristic of the OH8245 recurrent parent.

Tomato plants often exhibit isohydric (saver) and anisohydic (spender) responses to water deficit stress (Sade et al., 2012). A typical isohydric response involves a decline in g_*sw*_ before any adverse effects of water shortage arise in the canopy (Sade et al., 2012). In contrast, ansiohydric response involves a decline in leaf water potential and stomatal conductance proportional to soil moisture (Sade et al., 2012). Physiological data suggest that LA1141 behaves more like an isohydric plant because of its ability to maintain low canopy temperature, higher g_*sw*_, and lower VPD after 72 h of water deficit. In contrast, OH8245 appears to behave like an ansiohydric plant, a response that is associated with higher yields and biomass under moderate stress. This response of OH8245 is consistent with its previously described physiology compared to water deficit tolerant Mediterranean tomato germplasm (Galmés et al., 2011). In situations of water deficit that result in a plant reaching a permanent wilting point, anisohydric behavior may endanger plant survival (Sade et al., 2012). However, plant recovery after deficit irrigation was not evaluated in these studies.

### High-throughput thermal image analysis

Image-based estimations of canopy temperature in plants subjected to water deficit can serve as a proxy to plant physiological response. Interest in using canopy temperature as an indicator of plant stress has an extensive history (Jackson et al., 1981; Jones et al., 2002; Gerhards et al., 2016; Perera et al., 2020). Canopy temperature is directly proportional to stomatal conductance in many crops that are subjected to water deficit conditions (Jackson et al., 1988; Ramírez et al., 2016; Fukuda et al., 2018). Canopy temperature can be measured radiometrically using infrared thermometry and by extracting temperature from digital images (Blum et al., 1982; Babar et al., 2006; Gräf et al., 2021). Increased efficiencies achieved using infrared thermometry (Blum et al., 1982) or thermal images (thermography) (Ru et al., 2020; Vieira and Ferrarezi, 2021) enable high throughput data collection to characterize the physiological and genetic consequences of water-deficit stress in large populations or germplasm screens. Genetic variation for canopy temperature has been identified and used for plant improvement in both grain (Lopes et al., 2012) and vegetable crops (Prashar et al., 2013). For example, genetic variation for canopy temperature in runner beans and wheat is linked to maintaining stomatal conductance in water-limited environments (Fischer et al., 1998; Jones, 1999).

Genetic studies suggest tolerance to water deficit stress is a polygenic trait controlled by several small-effect QTLs (Sacco et al., 2013). Hundreds of genes can be activated or repressed in response to water deficit stress (Bray, 2004), making it hard to pinpoint which gene contributes to tolerance. An improved understanding of both the physiological traits and the genetic basis of plant response to water deficit may result in genetic gains in breeding programs by determining the proportion of genetic variation present in populations, estimating the heritability of traits associated with response to water deficit, and modeling environmental effects on traits of interest (Mir et al., 2012).

Generally, when plants experience water deficit stress, their stomata close as a strategy to conserve water. As stomatal conductance declines in response to this closure, leaf temperature can increase rapidly due to loss of evaporative cooling, and an increase in convective heat exchange (Jackson et al., 1988; Lambers et al., 2008). The physical phenomenon of stomatal closure in response to water deficit is inversely proportional to VPD, and the closure also leads to decreased transpiration and carbon assimilation (Lambers et al., 2008). Measuring plant canopy temperature under water deficit may, therefore, provide an efficient phenotyping alternative to physiological measurements of both stomatal conductance (Zhao et al., 2021) and VPD (Grossiord et al., 2020).

Assessment of canopy temperature provided an efficient measure of response to water deficit stress. Plants under water deficit stress generally have reduced g_*sw*_, limited transpiration, and increased canopy temperature. Therefore, canopy temperature was used to proxy these traits. Additionally, canopy temperature was used to phenotype and select tolerant BC_2_S_3_ families and recovered progenies that maintain higher g_*sw*_ and lower VPD under deficit irrigation. This suggested that indirect selection was possible with canopy temperature and turgor evaluations. The image analysis workflow was scalable to large populations and efficient for accurate phenotyping in the greenhouse. Water deficit stress evaluations of plants with images partitioned a higher proportion of the total variance into genetics, improved the objectivity of evaluations, and saved time.

Thermal image and IRT estimated canopy temperature are both effective phenotyping strategies, but IRT measures canopy temperature using single-point leaf measurements. These single-point measurements may not capture the entire gradient of temperature across the canopy (Figure 3). Temperature gradients across the canopy are likely why point measurements partition more variance into environmental factors and error relative to genetic factors (Table 1). Thermal image analysis also detected a greater range of differences in susceptible genotypes than the IRT. Additionally, the image-based analysis identified a canopy temperature’s QTL on the distal arm of chromosome 1 in our composite interval mapping study that was detected across seasonal environments but not detected with IRT (Table 2 and Figure 4). Canopy temperature measured by image-based methods may bring greater discrimination and sensitivity for selection and genetic analysis. Importantly, the thermal imaging workflow detected stress before the appearance of wilt symptoms (Supplementary Figure 4). Although thermal imaging was an effective method for trait evaluation in our studies, plant turgor ratings had the highest estimates of variance partitioned into genetic effects (Table 1). High-throughput phenotyping is promoted to reduce time, costs, and resources to screen populations in breeding programs (Cabrera-Bosquet et al., 2012; Fahlgren et al., 2015; Araus et al., 2018). However, visual evaluation of plant response to water deficit using a visual turgor index (Waterland et al., 2010) appears to be an effective method for evaluating germplasm.

### Genetics of water deficit tolerance derived from LA1141

Composite interval mapping helped discover chromosomal regions associated with low canopy temperature and higher turgor under water deficit stress (Figure 4). We found evidence for four QTLs, three of which were reproducible across environments and within the combined analysis. As expected, tolerance derived from LA1141 appears to be mediated by many loci and no large-effect QTLs were found. One interpretation of these results is that water deficit tolerance as measured by turgor and canopy temperature is genetically complex, with many QTL falling below the detection threshold. Genomic selection models offered a solution to the genetic complexity of water deficit stress tolerance because of their capacity to handle traits with many small-effect QTL (Crossa et al., 2017). The number of selections that possessed introgression from LA1141 that were associated with putative QTL and were discovered in the mapping study ranged from 15 to 40%, suggesting that we can select for putative QTL and make progress using an introgression strategy, even if the effects of allele substitutions and proportion of phenotypic variance explained are relatively low.

### Selection strategies

Phenotypic selection based on high throughput thermal image analysis *via* proximal sensing and genomic selection provides methods to improve response to deficit irrigation in progenies derived from the LA1141 × OH8245 families. Selection strategies based on canopy temperature and turgor also indirectly improved plant physiological response under water deficit measured by g_*sw*_ and VPD. Overall, prediction accuracy suggested that genomic selection alone may be an effective strategy for evaluating germplasm for tolerance to deficit irrigation as measured by low canopy temperature and high turgor. Additionally, using genomic selection in the future may save time spent phenotyping in additional generations during germplasm screens. Gain under selection was achieved for canopy temperature and turgor ratings. Selection models that incorporate low canopy temperature and visual plant ratings associated with high turgor have also indirectly selected lines with higher g_*sw*_ and lower VPD under deficit irrigation. Again, this suggests that our phenotyping methodology can be used to proxy plant physiological response and will potentially save time during future germplasm screens. In summary, phenotypic, genomic, and combined selection strategies have identified advanced lines with improved performance when grown under water deficit stress relative to randomly advanced lines. In our evaluations, improved performance was indicated by lower canopy temperature, higher turgor ratings, higher g_*sw*_, and lower VPD. These results provided a measure and confirmation of direct and indirect genetic gain toward water-deficit stress tolerance in our parent material, inbred backcross families, and selected progenies.

Water-deficit stress tolerance may also be selected for in-breeding populations using knowledge of marker-trait associations. Quantifying canopy temperature responses in large populations rapidly with objective and repeatable methods may improve QTL discovery. The genetic complexity of water deficit tolerance and the number of loci that may potentially be involved suggests that alternative strategies that estimate genome-wide effects to predict progeny performance have merit. Genomic selection (GS) (Heffner et al., 2010; Lorenz et al., 2011) changes the focus of analysis from identifying significant associations for QTL to estimating the effect of each marker across the genome. The sum of individual effects provides a genomic estimated breeding value for each family in the population. Our results confirm that GS strategies can be coupled with quantitative phenotyping to develop appropriate selection methods for traits associated with improved physiological status under water deficit. If adequate selection accuracies for complex traits can be achieved, GS has the potential to expedite genetic gain (Heffner et al., 2010; Cabrera-Bosquet et al., 2012). Selection indices developed in this study provide a framework to improve water deficit tolerance using both GS and phenotypic strategies.

### Agronomic and quality traits of selections

Acceptable yield, canopy width, and quality parameters were recovered in selections that were chosen based on phenotypic, genomic, and combined strategies. These results demonstrate that we can create selection indices to improve water-deficit tolerance in a recurrent parent background. Additionally, future crossing and evaluation are warranted. The fruit size of selections was small compared to the recurrent parent. Still, some selections yield well and have a canopy size that falls within the acceptable range for commercial processing tomatoes. The failure to recover acceptable fruit size is not surprising in an advanced BC_2_ population that used a small-fruited wild accession as the tolerant donor parent. Further crossing and selection will be needed to combine water-deficit stress tolerance and recurrent parent fruit size. However, traits associated with water deficit stress tolerance were successfully introgressed, and at the same time, important agronomic and quality traits associated with commercial processing tomatoes were also recovered.

## Conclusion

This work was initiated for the simultaneous introgression and discovery of tolerance to water deficit exhibited by the crop wild relative *S. galapagense* accession LA1141. A thermal image analysis workflow was developed for the population screens, provided an efficient measure of canopy temperature, and was a suitable proxy for physiological traits like g_*sw*_ and VPD. Analysis of canopy temperature using thermal images at 48 h of water deficit was able to predict turgor ratings, stomatal conductance, and vapor pressure deficit at 72 h water deficit stress. These results suggested that additional efficiencies based on time can be achieved for population evaluations. We were able to identify putative QTL derived from LA1141 associated with low canopy temperature and the ability to maintain high turgor in plants under water deficit. However, no large-effect QTLs were identified. Genomic and phenotypic selection indices offered a feasible strategy to recover tolerance in advanced lines despite the complexity of the trait. Additionally, applying both phenotype-based and genomic selection resulted in the recovery of the putative QTL at a higher-than-expected frequency. Although we successfully selected tomato lines tolerant to water deficit stress, we were unable to recover the fruit size for the direct commercial use of these selections. Finally, the germplasm created in these studies provides a resource for studying traits from LA1141, and we can use the advanced BC_2_S_5_ selections for future tomato improvement.

## Data availability statement

The datasets presented in this study can be found in online repositories. The names of the repository/repositories and accession number(s) can be found in the article/Supplementary material.

## Author contributions

SF and DF: conceptualization and experimental design. SF, JC, and JM: phenotyping. SF: high throughput pipeline development and analysis and writing. SF, KM, and DF: data analysis. SF, JC, and DF: population development. JM, KM, and DF: contribution to writing. All authors contributed to the article and approved the submitted version.

## Conflict of interest

The authors declare that the research was conducted in the absence of any commercial or financial relationships that could be construed as a potential conflict of interest.

## Publisher’s note

All claims expressed in this article are solely those of the authors and do not necessarily represent those of their affiliated organizations, or those of the publisher, the editors and the reviewers. Any product that may be evaluated in this article, or claim that may be made by its manufacturer, is not guaranteed or endorsed by the publisher.

## Footnotes

1
https://doi.org/10.5281/zenodo.5650152
