# Supplementary material for: Selection strategies to introgress water deficit tolerance derived from Solanum galapagense accession LA1141 into cultivated tomato
Source: Front Plant Sci. 2022 Jul 28;13:947538. doi: 10.3389/fpls.2022.947538 (PMC9366722; doi:10.3389/fpls.2022.947538)
Supplement: Supplementary file 1 [file Data_Sheet_1.PDF]

Supplementary Table S1| Percent of total variance estimates for thermal image estimated canopy temperature thresholding algorithms.

	Image thresholding algorithms[z]					
**Sources of variation[Y]**	**MEAN**	**Moments**	**Yen**	**Otsu**	**Default**	**Intermodes**
**GENOTYPE**	22.16	17.73	15.22	14.57	14.50	13.40
**ROW**	0.00	6.25	4.36	4.57	4.55	4.86
**COL**	2.80	29.26	32.18	29.64	29.81	28.96
**Residual**	75.04	46.77	48.24	51.22	51.14	52.78

[z] Thresholding algorithms "MEAN", "Moments", "Yen", "Otsu", "Default", and "Intermodes" calculated as described in Glasbey (1993). All thresholding algorithms are available in the ImageJ core package.

[y] GENOTYPE is represented by *S. galapagense* accession LA1141(N=9), *S. lycopersicum* OH8245 (N=9), BC_2S_5 inbred backcross lines (N=30, replicated three times). ROW and COLUMN were used as environmental terms to capture spatial variation across the greenhouse and each row by column location contained both replicated parental controls. Residual is experimental error.

Supplemental Table S2| Analysis of variance of water deficit tolerance traits for LA1141 and OH8245 during 72 hours of water deficit.

		Water deficit (hours) [x]			
**Effects** [z]	Df [y]	0	24	48	72
**Turgor rating (1-5)**					
Parents	1	NA	0.334	1.19e-05 ***	1.50e-15 ***
Block	2	NA	0.393	0.575	0.595
Residual	14				
**Canopy temperature (Image, °C)**					
Parents	1	0.385	0.085	0.049 *	NA
Block	2	0.886	0.033	0.322	NA
Residual	14				
**Canopy temperature (IRT, °C)**					
Parents	1	0.511	0.663	0.032 *	0.018 *
Block	2	0.352	0.939	0.993	0.379
Residual	14				
**Stomatal conductance (g_{sw}, mol $m^{-2}s^{-1}$)**					
Parents	1	0.104	0.071	0.6985	1.80e-07 ***
Block	2	0.409	0.2814	0.5217	0.061
Residual	14				
**Vapor pressure deficit (VPD, kPa)**					
Parents	1	0.2237	0.1098	0.5096	0.025 *
Block	2	0.5179	0.199	0.5062	0.122
Residual	14				
**Light adapted chlorophyll fluorescence (PhiPS2, 1-Fs⁄Fm)**					
Parents	1	0.2521	0.6156	0.09986	0.009 **
Block	2	0.5946	0.2217	0.48978	0.111
Residual	14				

[z] Parents include the *Solanum. galapagense* accession LA1141 donor parent (N=9) and the *S. lycopersicum* OH8245 recurrent parent (N=9). Block represents within experiment replication (three replicates in three blocks). Turgor is based on rating scale ranged from 1 to 5 (5=turgid, 4=soft to the touch, 3=beginning to wilt, 2=wilted with complete loss of turgor, and 1=dead) consistent with previous studies (Waterland et al., 2010). Canopy temperature was measured using a FLIRONE GEN3 iOS thermal camera (FLIR Systems Wilsonville, OR USA). Canopy temperature was also estimated using a handheld infrared thermometer (IRT) (Zhuhai JiDa Huapu Instrument Co., Hong Kong). Stomatal conductance to H_20 is g_{sw} mol m^{-2} s^{-1}. Vapor pressure deficit is VPD kPa at leaf temperature. Light adapted chlorophyll florescence is PhiPS2 1-$^{Fs}/_{FM}$. Stomatal conductance, vapor pressure deficit, and light adapted chlorophyll florescence was measured using the LI-600 porometer/fluorometer (LI-COR Bioscienes, Lincoln, NE, USA).

[y] Degrees of freedom.

[x] Water deficit (hours) represents no irrigation for a consecutive period of 0, 24, 48 and 72 hours.

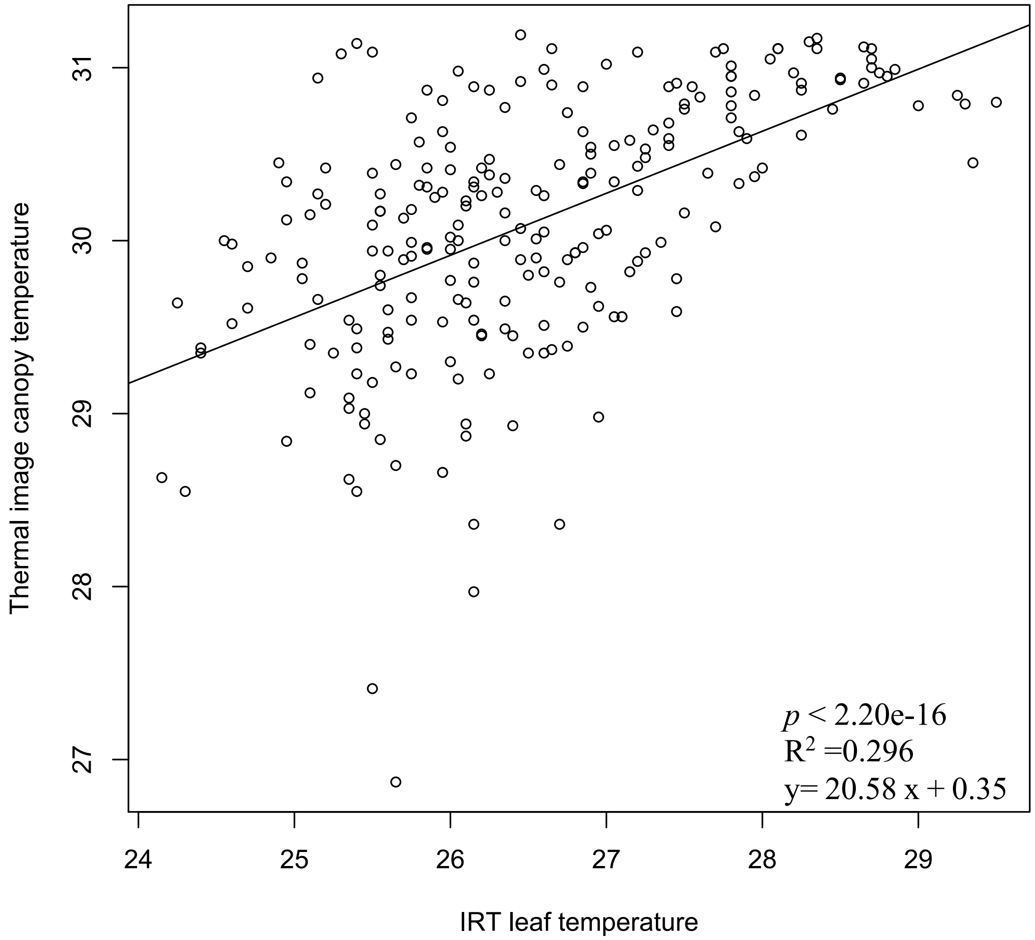

Supplementary Figure S3| Correlation between thermal images and infrared thermometer (IRT) estimated canopy temperature. Canopy temperature was measured with the images and the IRT simultaneously at maximum canopy temperature in the LA1141 × OH8245 BC_2S_3 families. The IRT and the thermal images were calibrated against a standard laboratory thermometer using an ice bath calibration method (Horwitz, 1999). All measurements took place between 10:00 and 12:00. Canopy temperature was estimated from images captured with the FLIRONE GEN3 iOS thermal camera (FLIR Systems Wilsonville, OR**).** IRT measurements were performed using a handheld infrared thermometer (Zhuhai JiDa Huapu Instrument Co., Hong Kong) and represent the average surface temperature of two, upper, fully expanded leaves.

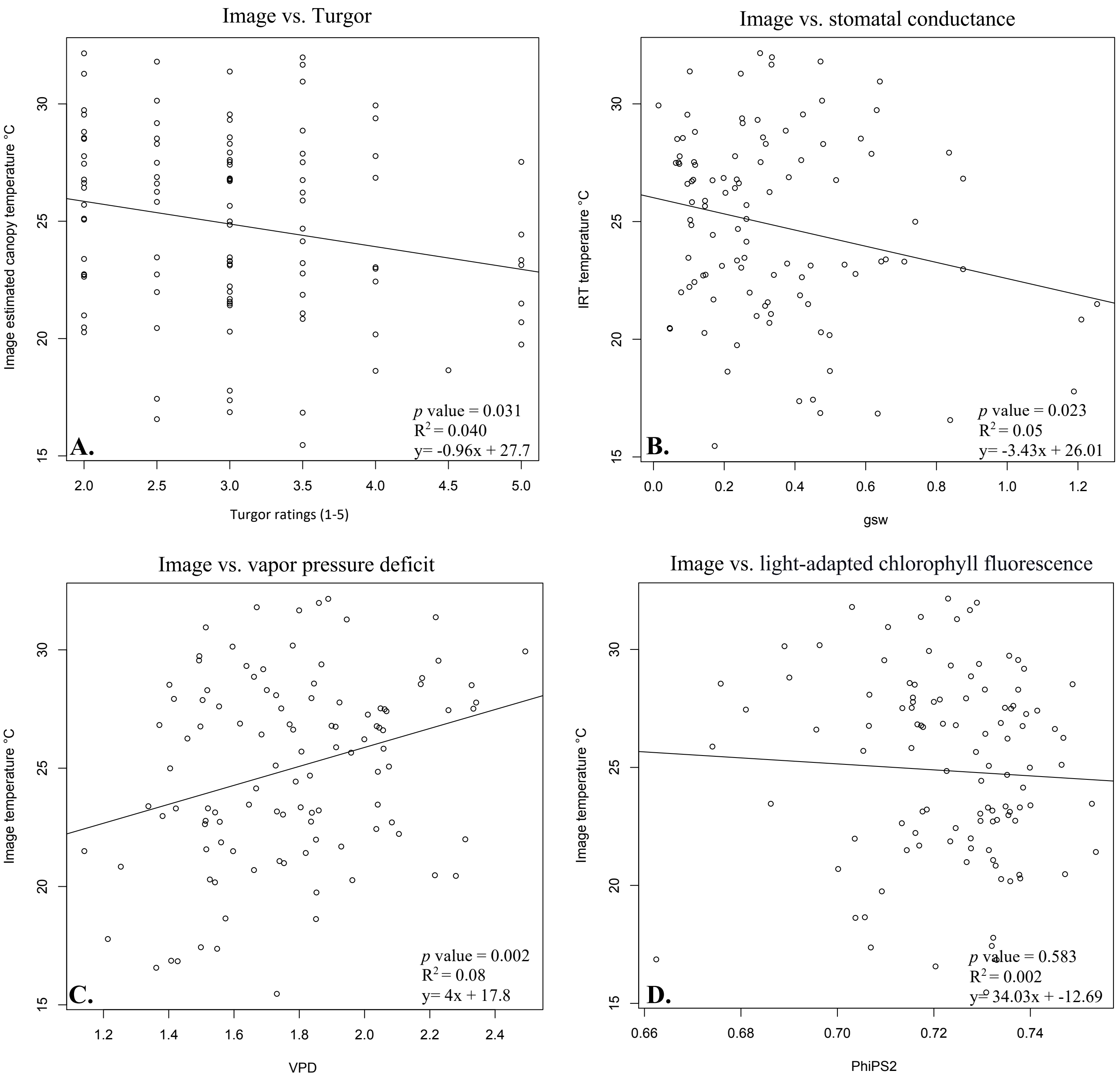

Supplementary Figure S4| Correlations of image-estimated canopy temperature (48 h water deficit stress). Image-based canopy temperature was regressed to **(A)** Turgor ratings, **(B)** stomatal conductance (g_{sw} m^{-2} s^{-1}), **(C)** vapor pressure deficit (VPD kPA), and **(D)** light-adapted chlorophyll fluorescence (PhiPS2 1-Fs/Fm). Images were captured with the FLIRONE GEN3 iOS thermal camera (FLIR Systems Wilsonville, OR). Turgor ratings ranging from 1 to 5 (5=turgid, 4=soft to the touch, 3=beginning to wilt, 2=wilted with complete loss of turgor, and 1=dead) as described previously (Waterland et al., 2010). Physiological measurements represented by stomatal conductance, vapor pressure deficit, and light-adapted chlorophyll fluorescence were taken with the LI-600 Porometer/ Fluorometer (LI-COR Bioscienes, Lincoln, NE). Turgor and physiological measurements were recorded at 72 h of water deficit stress.

Supplementary Table S5| Summary of greenhouse conditions in summer and fall LA1141 × OH8245 BC_2S_3 family water deficit stress germplasm screens.

IBC germplasm evaluation environment	Date/Time	Hours of withheld irrigation	HS; 66; Climate Temperature(°C)	HS; 66; Climate Humidity(%Rh)	HS; 66; OmniSensor PAR Light(µmol)	Notes
Summer	6/29/18 10:00	0	27.0	67	220	Water stress treatment began
Summer	6/29/18 10:30	0	27.2	66.7	264	
Summer	6/29/18 11:00	0	26.9	69.8	246	
Summer	6/29/18 11:30	0	26.8	70.6	285	
Summer	6/29/18 12:00	0	27.2	69.8	322	
Summer	**Mean**	0	**27.0**	**68.8**	**267.4**	
Summer	**Minimum**	0	**26.8**	**66.7**	**220.0**	
Summer	**Maximum**	0	**27.2**	**70.6**	**322.0**	
Summer	**Standard deviation**	0	**0.2**	**1.8**	**38.8**	
Summer	6/30/18 10:00	24	26.6	74	210	
Summer	6/30/18 10:30	24	26.9	73.7	256	
Summer	6/30/18 11:00	24	27.5	72.2	245	
Summer	6/30/18 11:30	24	27.6	72.6	258	
Summer	6/30/18 12:00	24	28.1	71.3	317	
Summer	**Mean**	24	**27.3**	**72.8**	**257.2**	
Summer	**Minimum**	24	**26.6**	**71.3**	**210.0**	
Summer	**Maximum**	24	**28.1**	**74.0**	**317.0**	
Summer	**Standard deviation**	24	**0.6**	**1.1**	**38.6**	
Summer	7/1/18 10:00	48	26.5	76.2	218	
Summer	7/1/18 10:30	48	27.2	75	262	
Summer	7/1/18 11:00	48	27.6	74.4	244	
Summer	7/1/18 11:30	48	28.2	73.5	281	
Summer	7/1/18 12:00	48	28.6	71.4	314	
Summer	**Mean**	48	**27.6**	**74.1**	**263.8**	
Summer	**Minimum**	48	**26.5**	**71.4**	**218.0**	
Summer	**Maximum**	48	**28.6**	**76.2**	**314.0**	
Summer	**Standard deviation**	48	**0.8**	**1.8**	**36.4**	

Summer	7/2/18 10:00	72	27.1	68.9	115	Day of maximum canopy temperature
Summer	7/2/18 10:30	72	26.4	75.5	221	
Summer	7/2/18 11:00	72	27.2	74.7	244	
Summer	7/2/18 11:30	72	27.5	74.4	280	
Summer	7/2/18 12:00	72	28.4	73.3	316	
Summer	**Mean**	72	**27.3**	**73.4**	**235.2**	
Summer	**Minimum**	72	**26.4**	**68.9**	**115.0**	
Summer	**Maximum**	72	**28.4**	**75.5**	**316.0**	
Summer	**Standard deviation**	72	**0.7**	**2.6**	**76.3**	
Summer	7/3/18 10:00	96	27.6	72.4	103	
Summer	7/3/18 10:30	96	27.4	73.4	106	
Summer	7/3/18 11:00	96	27.4	71.3	169	
Summer	7/3/18 11:30	96	27.2	74	202	
Summer	7/3/18 12:00	96	27.6	75.5	302	
Summer	**Mean**	96	**27.4**	**73.3**	**176.4**	
Summer	**Minimum**	96	**27.2**	**71.3**	**103.0**	
Summer	**Maximum**	96	**27.6**	**75.5**	**302.0**	
Summer	**Standard deviation**	96	**0.2**	**1.6**	**81.9**	
Summer	7/4/18 10:00	120	28.1	74.6	217	
Summer	7/4/18 10:30	120	28.2	77.3	257	
Summer	7/4/18 11:00	120	28.8	76.3	248	
Summer	7/4/18 11:30	120	29.7	70.7	236	
Summer	7/4/18 12:00	120	30.1	69.4	364	
Summer	**Mean**	120	**28.9**	**73.7**	**264.4**	
Summer	**Minimum**	120	**28.1**	**69.4**	**217.0**	
Summer	**Maximum**	120	**30.1**	**77.3**	**364.0**	
Summer	**Standard deviation**	120	**0.9**	**3.5**	**57.7**	
Summer	7/5/18 10:00	144	27.8	76.3	214	Day of maximum wilt (lowest turgor)
Summer	7/5/18 10:30	144	27.8	76.8	256	
Summer	7/5/18 11:00	144	28.2	75.3	247	
Summer	7/5/18 11:30	144	29.0	74.3	280	
Summer	7/5/18 12:00	144	29.6	72.8	257	

Summer	**Mean**	144	**28.5**	**75.1**	**250.8**	
Summer	**Minimum**	144	**27.8**	**72.8**	**214.0**	
Summer	**Maximum**	144	**29.6**	**76.8**	**280.0**	
Summer	**Standard deviation**	144	**0.8**	**1.6**	**23.9**	
Fall	11/2/18 10:00	0	26.4	38.5	301	Water stress treatment began
Fall	11/2/18 10:30	0	26.9	35.4	322	
Fall	11/2/18 11:00	0	26.8	33.1	342	
Fall	11/2/18 11:30	0	26.8	33	262	
Fall	11/2/18 12:00	0	26.3	33.9	227	
Fall	**Mean**	0	26.7	34.8	290.8	
Fall	**Minimum**	0	26.3	33.0	227.0	
Fall	**Maximum**	0	26.9	38.5	342.0	
Fall	**Standard deviation**	0	0.3	2.3	46.4	
Fall	11/3/18 10:00	24	25.3	35.5	245	
Fall	11/3/18 10:30	24	26.3	33.5	278	
Fall	11/3/18 11:00	24	27.2	30.4	662	
Fall	11/3/18 11:30	24	26.9	27	616	
Fall	11/3/18 12:00	24	27.1	24.6	313	
Fall	**Mean**	24	26.5	30.2	422.8	
Fall	**Minimum**	24	25.3	24.6	245.0	
Fall	**Maximum**	24	27.2	35.5	662.0	
Fall	**Standard deviation**	24	0.8	4.5	199.5	
Fall	11/4/18 10:00	48	27.3	29.8	439	Day of maximum canopy temperature
Fall	11/4/18 10:30	48	26.8	28.1	520	
Fall	11/4/18 11:00	48	26.5	27.8	206	
Fall	11/4/18 11:30	48	26.5	29	189	
Fall	11/4/18 12:00	48	26.9	28.6	201	
Fall	**Mean**	48	26.8	28.7	311.0	
Fall	**Minimum**	48	26.5	27.8	189.0	
Fall	**Maximum**	48	27.3	29.8	520.0	
Fall	**Standard deviation**	48	0.3	0.8	156.6	

Fall	11/5/18 10:00	72	26.9	37.7	284	
Fall	11/5/18 10:30	72	27.2	37.1	310	
Fall	11/5/18 11:00	72	26.6	37.7	252	
Fall	11/5/18 11:30	72	27.8	36.1	409	
Fall	11/5/18 12:00	72	27.1	36.1	351	
Fall	**Mean**	72	27.1	36.9	321.2	
Fall	**Minimum**	72	26.6	36.1	252.0	
Fall	**Maximum**	72	27.8	37.7	409.0	
Fall	**Standard deviation**	72	0.4	0.8	61.0	
Fall	11/6/18 10:00	96	26.8	40.2	293	
Fall	11/6/18 10:30	96	27.0	36.2	401	
Fall	11/6/18 11:00	96	27.1	34.4	681	
Fall	11/6/18 11:30	96	26.7	32.7	217	
Fall	11/6/18 12:00	96	26.4	34.7	185	
Fall	**Mean**	96	26.8	35.6	355.4	
Fall	**Minimum**	96	26.4	32.7	185.0	
Fall	**Maximum**	96	27.1	40.2	681.0	
Fall	**Standard deviation**	96	0.3	2.8	200.1	
Fall	11/7/18 10:00	120	28.2	24.9	662	
Fall	11/7/18 10:30	120	27.8	23.8	423	
Fall	11/7/18 11:00	120	27.2	23.6	443	
Fall	11/7/18 11:30	120	26.7	22.7	382	
Fall	11/7/18 12:00	120	26.5	23.1	188	
Fall	**Mean**	120	27.3	23.6	419.6	
Fall	**Minimum**	120	26.5	22.7	188.0	
Fall	**Maximum**	120	28.2	24.9	662.0	
Fall	**Standard deviation**	120	0.7	0.8	169.1	
Fall	11/8/18 10:00	144	28.4	24.1	637	Day of maximum wilt (lowest turgor)
Fall	11/8/18 10:30	144	27.2	24.5	266	
Fall	11/8/18 11:00	144	26.8	24.5	494	
Fall	11/8/18 11:30	144	26.6	24	375	
Fall	11/8/18 12:00	144	25.5	25.4	185	
Fall	**Mean**	144	26.9	24.5	391.4	
Fall	**Minimum**	144	25.5	24.0	185.0	

Fall	**Maximum**	144	28.4	25.4	637.0
Fall	**Standard deviation**	144	1.0	0.6	179.9

Supplemental Table S6| Analysis of variance of water deficit tolerance traits for BC_2S_3 families in summer and fall germplasm screens.

Effects[z]	Turgor rating[y]	Canopy temperature (°C) [x]	Pot weight loss (g) (evapotranspiration)[w]
		p values [v]	
Genotype	< 2.20e-16 ***	8.176e-07 ***	0.461
Environment	< 2.20e-16 ***	0.736	3.42e-08 ***
Environment × Row	0.007	0.371	0.089
Environment × Column	0.0002 ***	5.05E-14 ***	0.732

[z] Genotype represents the LA1141(N=36), the OH8245 (N=36), and the LA1141 × OH8245 BC_2S_3 families (N=160). Environment is summer (July) and fall (November) seasonal environments during germplasm screens. The environmental terms Row and Column were used to capture spatial variation within the greenhouse across the air movement between cooling pads and fans and light gradients. The Row and Column interactions with environment are spatial variation within each experiment.

[y] Turgor is based on a rating scale ranged from 1 to 5 (5=turgid, 4=soft to the touch, 3=beginning to wilt, 2=wilted with complete loss of turgor, and 1=dead) consistent with previous studies (Waterland et al., 2010).

[x] Canopy temperature measured as whole plant canopy temperature (°C) using a FLIRONE GEN3 iOS thermal camera (FLIR Systems Wilsonville, OR USA).

[w] Water loss through evapotranspiration during deficit irrigation treatments estimated as pot weight loss.

[v] A fully parameterized model was compared to a model with a single term dropped using a likelihood ratio test based on a chi-square distribution (Snijders and Bosker 2012). A significant p-value was interpreted as evidence that the parameter dropped was important to the fit of the model and Bayesian Information Content (BIC) values were used to confirm that the full model provided a better fit to confirm the significance of genetic and environmental terms.

Supplemental Table S7| Analysis of variance of water deficit tolerance traits for comparisons of selection strategies.

**Effects** [z]	**Df** [y]	***p* value**
**Turgor rating**		
Selection strategy	3	0.004 **
Residuals	26	
**Canopy temperature**		
Selection strategy	3	0.006 **
Residuals	26	
**Stomatal conductance (g_{sw} mol $m^{-2}s^{-1}$)**		
Selection strategy	3	0.026 *
Residuals	26	
**Vapor pressure deficit (VPD kPa)**		
Selection strategy	3	0.046 *
Residuals	26	

[Z] Selection strategy represents the selection categories based on GEBVs (GS, N=8), LA1141 × OH8245 BC_2S_3 canopy temperature and wilt BLUPs (Pheno, N=9), a combination of the two (GS + Pheno, N=3), and randomly advanced lines (Random, N=10). Treatments were replicated three times. Plant turgor is based on a rating scale ranging from 1 to 5 (5=turgid, 4=soft to the touch, 3=beginning to wilt, 2=wilted with complete loss of turgor, and 1=dead) consistent with previous studies (Waterland et al., 2010). Canopy temperature was measured as whole plant canopy temperature (°C) using a FLIRONE GEN3 iOS thermal camera (FLIR Systems Wilsonville, OR USA). Stomatal conductance is g_{sw} mol m^{-2} s^{-1}. Vapor pressure deficit is VPD kPa at leaf temperature. Values are reported at the time point where they reach their maximums. Stomatal conductance and vapor pressure deficit was measured using the LI-600 porometer/fluorometer (LI-COR Biosciences, Lincoln, NE, USA).

[Y] Degrees of freedom.

References

Glasbey, C. A. (1993). An Analysis of Histogram-Based Thresholding Algorithms. In *Graphical Models and Image Processing* (Vol. 55, Issue 6, pp. 532–537). https://doi.org/10.1006/gmip.1993.1040

Horwitz, J. W. (1999). Water at the ice point: a useful quasi-blackbody infrared calibration source. *Applied Optics*, *38*(19), 4053. https://doi.org/10.1364/ao.38.004053

Snijdgers, T. A. B., Bosker, R. J. (2012). *Multilevel analysis : an introduction to basic and advanced multilevel modeling* (2nd). Sage Publications.

Waterland, N. L., Campbell, C. A., Finer, J. J., & Jones, M. L. (2010). Abscisic acid application enhances drought stress tolerance in bedding plants. *HortScience*, *45*(3), 409–413. https://doi.org/10.21273/hortsci.45.3.409
